# An integrated taxonomic and conservation assessment of *Glauconycteris* (Chiroptera, Vespertilionidae) in Cameroon, with the description of two new species from the Northwestern Congolian Lowland Forest

**DOI:** 10.3897/zookeys.1282.183038

**Published:** 2026-06-15

**Authors:** Aicha Gomeh-Djame, Paul J. J. Bates, Javier Juste, Marcela Suarez-Rubio, Laura Torrent, Juan Luis García-Mudarra, Eugenie Agodigo Ayangma, Félécité Yettore, Charles Félix Bilong Bilong, Eric-Moise Bakwo-Fils

**Affiliations:** 1 Laboratory of Zoology, Department of Biology and Animal Physiology, Faculty of Science, University of Yaounde 1, Yaounde, Cameroon Department of Evolutionary Ecology, Estación Biológica de Doñana (CSIC) Seville Spain; 2 Harrison Institute, Tremough Innovation Centre, Tremough Campus, Penryn, Cornwall, TR10 9TA, UK Faculty of Science, University of Yaounde 1 Yaounde Cameroon; 3 Centre for Ecology and Conservation, Faculty of Environment, Science and the Economy, Penryn Campus, University of Exeter, Cornwall, TR10 9FE, UK Faculty of Agronomy and Agricultural Sciences, University of Douala Douala Cameroon; 4 Department of Evolutionary Ecology, Estación Biológica de Doñana (CSIC), 41092, Seville, Spain Higher Institute of Agriculture, Forestry, Water and the Environment (HIAFWE), University of Ebolowa Ebolowa Cameroon; 5 CIBER of Epidemiology and Public Health, CIBERESP, Madrid, Spain CIBER of Epidemiology and Public Health Madrid Spain; 6 Institute of Zoology, Department of Ecosystem Management, Climate and Biodiversity, BOKU University, Gregor-Mendel-Strasse 33, 1180 Vienna, Austria Faculty of Sciences, University of Maroua Maroua Cameroon; 7 BiBio (Biodiversity and Bioindicators Research Group), Natural Sciences Museum of Granollers, 08402, Granollers, Spain Department of Ecosystem Management, Climate and Biodiversity, BOKU University Vienna Austria; 8 Department of Forestry, Faculty of Agronomy and Agricultural Sciences, University of Douala, Douala B.P. 2701, Cameroon Harrison Institute, Tremough Innovation Centre Penryn United Kingdom; 9 Department of Biological Sciences, Faculty of Sciences, University of Maroua, Maroua, Cameroon Faculty of Environment, Science and the Economy, Penryn Campus, University of Exeter Cornwall United Kingdom; 10 Higher Institute of Agriculture, Forestry, Water and the Environment (HIAFWE), University of Ebolowa, Ebolowa, Cameroon BiBio (Biodiversity and Bioindicators Research Group), Natural Sciences Museum of Granollers Granollers Spain

**Keywords:** Conservation status, cryptic diversity, forest dependent bats, Guineo-Congolian region, integrative taxonomy, molecular phylogenetics, Chauves-souris des forêt, diversité cryptique, phylogénie moléculaire, région guinéo-congolaise, statut de conservation, taxonomie intégrative

## Abstract

The genus *Glauconycteris* (Vespertilionidae) comprises small, forest-dependent bats distributed across sub-Saharan Africa. Despite its distinctive morphology, the genus remains poorly represented in collections and its taxonomy unresolved. During recent field surveys in southeastern Cameroon, ten *Glauconycteris* specimens were collected from the Northwestern Congolian Lowland Forest, eight from Lobéké National Park (LNP) and two from the Dja Biosphere Reserve (DBR). Morphometric and molecular analyses assigned these specimens to eight species, including representatives of three species groups (*poensis*, *beatrix*, and *humeralis*). One species, *G. superba*, is recorded for the first time from Cameroon and two are described herein as new to science, *Glauconycteris baka***sp. nov**. and *Glauconycteris lobeke***sp. nov**. These findings increase the total number of recognised *Glauconycteris* species to 15 and confirm that *G. beatrix*, as previously understood, represents a species complex, revealing further cryptic diversity within the genus. By combining taxonomic, ecological, and distributional data, our results indicate that most members of the genus tolerate moderate habitat degradation and that all 11 species recorded from Cameroon occur within or adjacent to at least one protected area. This study provides the first integrated taxonomic assessment of *Glauconycteris* in Cameroon, refines species distributions, and contributes additional ecological data for three species (*G. gleni*, *G. egeria*, and *G. curryae*) currently listed as Data Deficient on the IUCN Red List. Our findings underscore the importance of continued sampling in the Guineo-Congolian region to better inform the conservation of Africa’s forest-dependent bats.

## Abstract (fr)

Le genre *Glauconycteris* (Vespertilionidae) comprend des petites chauves-souris de forêt réparties en Afrique subsaharienne. En dépit de sa morphologie distinctive, ce genre est peu représenté dans les collections et sa taxinomie demeure non résolue. Des récents inventaires de terrain menés dans le sud-est du Cameroun, dix spécimens de *Glauconycteris* ont été collectés dans la forêt de basse altitude nord-ouest congolaise, dont huit dans le Parc National de Lobéké (PNL) et deux dans la Réserve de Biosphère du Dja (RBD). Les analyses morphométriques et moléculaires ont permis d’attribuer ces spécimens à huit espèces appartenant à trois groupes d’espèces (*poensis*, *beatrix* et *humeralis*). Une espèce, *G. superba*, est signalée pour la première fois au Cameroun et deux autres sont décrites ici comme nouvelles pour la science, *Glauconycteris baka***sp. nov**. et *Glauconycteris lobeke***sp. nov**. Ces découvertes portent à 15 le nombre total d’espèces reconnues au sein du genre *Glauconycteris* et confirment que *G. beatrix*, telle que précédemment définie, représente un complexe d’espèces, révélant ainsi une diversité cryptique supplémentaire au sein du genre. En intégrant des données taxonomiques, écologiques et de distribution, nos résultats indiquent que la plupart des représentants du genre tolèrent une dégradation modérée de l’habitat et que les 11 espèces recensées au Cameroun sont présentes à l’intérieur ou à proximité d’au moins une aire protégée. Cette étude constitue la première évaluation taxonomique intégrée du genre *Glauconycteris* au Cameroun, précise la distribution des espèces et apporte des données écologiques complémentaires sur trois espèces (*G. gleni*, *G. egeria* et *G. curryae*) actuellement classées dans la catégorie « Données insuffisantes » de la Liste rouge de l’UICN. Nos résultats soulignent l’importance de poursuivre des inventaires dans la région guinéo-congolaise afin de mieux orienter la conservation des chauves-souris de forêt d’Afrique.

## Introduction

The family Vespertilionidae is the most species-rich and widely distributed of all bat families (Moratelli et al. 2019). In Africa, vespertilionids account for more than one-third of known bat species (Monadjem et al. 2024), with new taxa described almost annually (e.g. Monadjem et al. 2020b; Grunwald et al. 2023; Juste et al. 2023; Torrent et al. 2025b). Among this megadiverse group, the genus *Glauconycteris* Dobson, 1875 is typically found near water and confined to sub-Saharan tropical forests and savannah woodlands (Happold and Happold 2013; Hassanin et al. 2018; ACR 2024). Like many of sub-Saharan Africa’s 266 bat species, *Glauconycteris* are infrequently captured and poorly represented in scientific collections (Monadjem et al. 2024). Of the 13 species recognised by Simmons and Cirranello (2025), three are known from fewer than ten localities (*G. atra*Hassanin et al. 2018, *G. gleni* Peterson & Smith, 1973, *G. superba* Hayman, 1939), and two only from their holotype (*G. kenyacola* Peterson, 1982, *G. machadoi* Hayman, 1963) (Table 1).

**Table 1.** Character matrix of all *Glauconycteris* species, arranged in descending order of greatest skull length (GSL). Measurements from Happold and Happold (2013), Hassanin et al. (2018) and this study. Number of localities in Africa and Cameroon based on Monadjem et al. (2024), Torrent et al. (2025a), and this study.

Species (IUCN status) Number of localities in Africa/Cameroon Key characters	FA, GSL, C-M^3^ measurements (mm)	Wings and uropatagium colour and reticulation. Pelage colour	Ear shape; tragus shape. Baculum shape	Dorsal profile of skull; upper inner incisor (I^2^)
*G. superba* (LC) Locs: 8/1 (Fig. 11A). Pelage colour; size	FA: 45-48 GSL: 16.2–16.5 C-M^3^: 5.8–6.2	Wings dark, not reticulated. Pelage black with creamy-white patches and stripes (Fig. 8A)	Ears subrectangular; tragi broad, rounded (Fig. 4A). Baculum: inverted U-shape (Fig. 5A)	Profile strongly concave (Fig. 10A). I^2^ unicuspid
*G. gleni* (DD) Locs: 4/2 (Fig. 11A). Pelage, wing, and ear colour	FA: 38-42 GSL: 13.9–14.6 C-M^3^: 4.5–4.9	Wings pale; uropatagium reticulated. Pelage pale greyish fawn above, white below (Fig. 8B)	Ears subrectangular with pronounced margins; tragi broad, rounded (Fig. 4B). Baculum: arrowhead, basal lobes deflected downwards (Fig. 5B)	Profile weakly concave (Fig. 10B). I^2^ weakly bicuspid/ unicuspid
*G. variegata* (LC) Locs: 113/3 (Fig. 11A). Wing reticulation; pelage colour	FA: 38-45 GSL: 13.6–14.5 C-M^3^: 4.7–5.2	Wings pale, heavily reticulated (Fig. 8C). Pelage creamy buff to yellowish fawn (Fig. 8C)	Ears rounded; tragi broad, rounded (Fig. 4C). Baculum: arrowhead, size varies (Fig. 5C)	Profile concave/weakly concave (Fig. 10C). I^2^ unicuspid/ bicuspid
*G. machadoi* (DD) Locs: 1/0. Wing reticulation; pelage colour	FA: 46 GSL: 14.0 C-M^3^: 5.2	Wings dark brown, heavily reticulated. Pelage dark chestnut brown above, paler below	Ears rounded; tragi probably comparable to *G. variegata*. Baculum not described	Profile weakly concave. I^2^ unicuspid
*G. alboguttata* (LC) Locs: 16/6 (Fig. 11B). Ear shape; pelage colour	FA: 38-42 GSL: 13.0–13.5 C-M^3^: 4.4–4.7	Wings dark brown, not reticulated. Pelage dark brown above; pale shoulder spot and flank stripe (Hassanin et al. 2018: fig. 6)	Ears rounded (contrast to *G. egeria*); tragi broad, rounded (Fig. 4D). Baculum not described	Profile slightly concave (Fig. 10D). I^2^ bicuspid
*G. argentata* (LC) Locs: 63/13 (Fig. 11B). Pelage colour; size; baculum	FA: 39-44 GSL: 12.1–13.3 C-M^3^: 4.0–4.5	Wings pale, faintly reticulated (Fig. 8D). Pelage variable, pale fawn to grey-brown above and below; paler flank stripe usually present; no shoulder spot (Fig. 8D)	Ears rounded, pale; tragi broad, rounded (Fig. 4E). Baculum: arrowhead (Fig. 5D)	Profile weakly concave (Fig. 9A). I^2^ unicuspid/ bicuspid
*G. kenyacola* (DD) Locs: 1/0. Pelage colour	FA: 41 GSL: 12.8 C-M^3^: 4.2	Wings ochraceous buff, some reticulation. Pelage sepia-brown; whitish markings on nose and base of each ear	Ears rounded; tragi not described. Baculum not described	Profile weakly concave. I^2^ unicuspid
*G. egeria* (DD) Locs: 13/6 (Fig. 11C). Ear shape, colour; pelage colour	FA: 37-39 GSL: 12.0–13.0 C-M^3^: 4.1–4.5	Wings dark brown, not reticulated. Pelage dark brown/black; pale shoulder spot and flank stripe (Hassanin et al. 2018: fig. 6)	Ears subrectangular with pale border; tragi broad, rounded (Fig. 4F). Baculum not described	Profile concave/weakly concave (Fig. 9B). I^2^ weakly bicuspid
*G. atra* (na) Locs: 3/0. Pelage colour; size	FA: 34.5–37.8 GSL: 12.1–12.6 C-M^3^: 4.2–4.5	Wings blackish brown, not reticulated. Pelage blackish brown; no pale shoulder spot or flank stripe.	Ears rounded; tragi straight/slightly concave anteriorly, convex posteriorly. Baculum not described	Profile concave. I^2^ strongly bicuspid
*G. lobeke* sp. nov. (na) Locs: 4/2 (Fig. 11C). Pelage colour; skull profile; baculum	FA: 35.8, 35.9 GSL: 12.2, 12.3 C-M^3^: 4.2–4.3	Wings mid-brown, not reticulated. Pelage sepia/darker brown; pale shoulder spot; no flank stripe (Fig. 3A)	Ears rounded; tragus rounded (Fig. 4G). Baculum: inverted V -shape (Fig. 5E)	Profile strongly concave (Fig. 6A). I^2^ bicuspid (Fig. 7A)
*G. curryae* (DD) Locs: 18/7 (Fig. 11C). Pelage colour; skull profile; baculum	FA: 34-38 GSL: 11.7–12.3 C-M^3^: 4.0–4.2	Wings dark brown, not reticulated. Pelage orange/reddish brown above, slightly paler below; no shoulder spot or flank stripe (Fig. 8E)	Ears rounded; tragi almost straight (Fig. 4H). Baculum: inverted Y-shape (Fig. 5F)	Profile strongly concave (Fig. 9C). I^2^ strongly bicuspid
*G. poensis* (LC) Locs: 65/2 (Fig. 11D). Pelage colour; skull profile	FA: 32-41 GSL: 10.8–13.0 C-M^3^: 4.0–4.8	Wings dark brown, faintly reticulated. Pelage greyish to brown (Hassanin et al. 2018: fig. 6); pale shoulder spot and flank stripe (either may be absent)	Ears rounded; tragi rounded (Happold and Happold 2013: fig. 124e). Baculum: inverted V-shape, variably developed (Happold and Happold 2013: fig. 128e, f)	Profile strongly concave (Fig. 9D). I^2^ weakly bicuspid
*G. beatrix* (LC) Locs: 40/7 (Fig. 11D). Pelage colour; skull profile; baculum	FA: 35-42 GSL: 10.6–12.0 C-M^3^: 3.6–4.6	Wings dark brown, not reticulated. Pelage sepia to darker brown; usually faint white shoulder spot; no flank stripe (Fig. 3B)	Ears rounded; tragi straight anteriorly (Fig. 4I). Baculum: inverted V-shape (Fig. 5G)	Profile concave/weakly concave (Fig. 6B/C). I^2^ strongly bicuspid (Fig. 7B)
*G. humeralis* (DD) Locs: 16/0. Pelage colour; skull profile; baculum	FA: 35-41 GSL: 10.8–12.0 C-M^3^: 3.6–4.2	Wings dark brown, not reticulated. Pelage dark sepia brown; conspicuous pale shoulder spot; no flank stripe	Ears rounded; tragi straight to slightly convex anteriorly (Fig. 4J). Baculum: peg-shaped (Happold and Happold 2013: fig. 128d) but V-shaped in Fig. 5H - see text	Profile concave/strongly concave (Fig. 6D). I^2^ strongly bicuspid (Fig. 7C)
*G. baka* sp. nov. (na) Locs: 4/1. (Fig. 11D). Pelage colour; skull profile; baculum	FA: 34.2 GSL: 10.7 C-M^3^: 3.7	Wings dark brown/ black, not reticulated. Pelage dark brown; pale shoulder spot; no flank stripe (Fig. 3C)	Ears rounded; tragi narrow, anterior straight anteriorly (Fig. 4K). Baculum: inverted Y-shape, expanded basal lobes (Fig. 5I)	Profile slightly concave (Fig. 6E). I^2^ bicuspid (Fig. 7D)

The genus *Glauconycteris* was originally described as a subgenus of *Chalinolobus* but was elevated to full generic rank by de Winton (1901), with *Glauconycteris* restricted to Africa and *Chalinolobus* to Australasia. Subsequent authors disagreed on its status, some retaining it as a subgenus (Smithers 1983; Koopman 1993, 1994) and others recognising it as a distinct genus (Ellerman et al. 1953; Hayman and Hill 1971; Peterson 1982). Hill and Harrison (1987) highlighted the distinctive morphology of the *Glauconycteris* baculum, further supporting its separation at the generic level. Later molecular studies corroborated this conclusion, although not all recognised species were included in those analyses (Hoofer and Van Den Bussche 2003; Roehrs et al. 2010; Roehrs et al. 2011). The complex taxonomic history of *Glauconycteris* has been reviewed in detail by Happold and Happold (2013), Hassanin et al. (2018), and the African Chiroptera Report (ACR 2024).

During the past two decades, molecular phylogenetics has profoundly reshaped the classification of African vespertilionid bats (e.g. Koubínová et al. 2013; Hassanin et al. 2018; Demos et al. 2018, 2020, 2024; Hutterer et al. 2019; Monadjem et al. 2021), revealing unexpected cryptic diversity and previously unrecognised evolutionary relationships across the continent. In the most comprehensive analysis of *Glauconycteris* to date, Hassanin et al. (2018) confirmed its monophyly using mitochondrial and nuclear markers and identified two principal lineages: one comprising *G. variegata* (Tomes, 1861) and *G. superba*, and the second containing all remaining species. Within this latter lineage, three well-supported clades were recognised: (i) the *poensis* group (*G. poensis* (Gray, 1842), *G. alboguttata* J.A. Allen, 1917, *G. argentata* (Dobson, 1875), and *G. egeria* Thomas, 1913); (ii) the *beatrix* group, comprising *G. beatrix* Thomas, 1901, with two distinct lineages (a) from Cameroon/Central African Republic (hereafter CAR) and (b) the Democratic Republic of Congo (hereafter DRC), together with *G. curryae* Eger & Schlitter, 2001; and (iii) the more complex *humeralis* group, which included *G. humeralis* J.A. Allen, 1917, the newly described *G. atra*, and two additional, well-supported but undescribed lineages referred to as *G. cf. humeralis* (from CAR) and *G. cf. beatrix* (from Côte d’Ivoire). Since that study, no further molecular work has focused specifically on *Glauconycteris*.

During recent field surveys in southeastern Cameroon, ten specimens of *Glauconycteris* were collected from the Northwestern Congolian Lowland Forest, including Lobéké National Park and the Dja Biosphere Reserve. These specimens were examined using an integrative approach combining morphological and molecular data. The aims of this study are to assess the taxonomic status of *Glauconycteris* populations in this region, evaluate their phylogenetic relationships within the genus, and describe newly identified taxa and their conservation significance.

## Materials and methods

### Study sites

**Figure 1.**
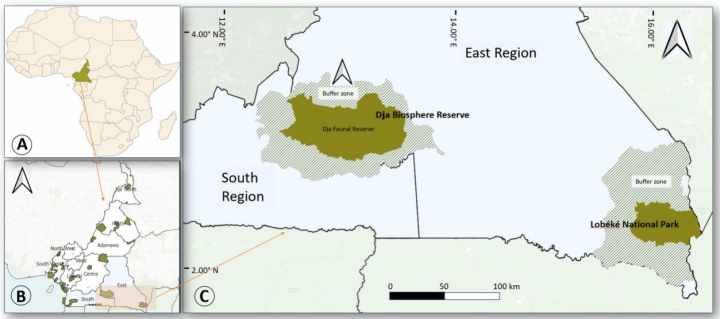
**A**. Map of Africa showing Cameroon (green). **B, C**. Maps of South and East Regions with Dja Biosphere Reserve (DBR), comprising Dja Faunal Reserve and its buffer zone and Lobéké National Park (LNP) and its buffer zone.

Fieldwork was conducted in southeastern Cameroon, primarily in Lobéké National Park (hereafter LNP; 2°18'N, 15°54'E; Fig. 1). Established in 2001, LNP forms part of the Trinational de la Sangha landscape, which also includes Dzangha-Sangha National Park in Central African Republic and the Nouabalé-Ndoki National Park in Republic of Congo (IUCN 2012; KBA 2025). It covers 2,178.5 km^2^ at elevations of ca 330–750 m a.s.l. and is characterised by semi-evergreen lowland forest with swamp forest and seasonally flooded grasslands (Dowsett-Lemaire and Dowsett 2000). The climate is Guinean equatorial with bimodal rainfall: two dry seasons (November–March and June–August) and two rainy seasons (March–June and August–November); mean annual rainfall of 1,570–1,800 mm (Olivry 1986; WWF 2022). Sampling was conducted both within the protected core of LNP and in the surrounding buffer zones. This buffer zone (5960 km^2^) includes Forestry Management Units (FMUs), characterised by selectively logged forest, which are embedded within a larger area of community-managed hunting zones (ZICGCs), comprising a mosaic of secondary forest, agricultural land and settlements (Lambini et al. 2019; Yuh et al. 2020). Habitat categories were used to distinguish between relatively intact and anthropogenically modified environments in subsequent analyses.

Additional material was obtained from the Dja Biosphere Reserve (hereafter DBR; 3°07'N, 13°03'E; Fig. 1), which includes the Dja Faunal Reserve (ca 5,260 km^2^) and its buffer zone. The latter comprises Forestry Management Units, settlements, community forests, some rubber plantations, and a hydroelectric dam with reservoir (Bruce et al. 2018). The reserve is bounded on three sides by the Dja River and consists of generally flat terrain at elevations of 400–800 m a.s.l., supporting lowland, dense semi-evergreen forest with swamp and riverine habitats within a transitional zone between Atlantic coastal and Congolian forest formations (MINFOF and IUCN 2015; Atagana et al. 2021; Munjeb et al. 2021; World Heritage Datasheet 2025). The climate is bimodal with two dry seasons (mid-November–March and June–August) and two rainy seasons (mid-March–May and August–mid-November); mean annual rainfall is ~ 1,570 mm (Bruce et al. 2018); temperatures remain relatively stable throughout the year (Vande Weghe 2004).

### Bat sampling

Bat sampling in LNP (2023–2024) spanned one rainy and three dry seasons. Effort was distributed across three distinct forest management zones (intact forest, FMUs, and ZICGCs), with 16–19 nights of sampling per zone (52 nights total). In DBR, a 10-night bat survey was undertaken in Messamena in the long dry season (November–December 2022). This complemented the earlier surveys of Atagana et al. (2021). In both LNP and DBR, mist nets, ranging from 6–18 m in length, 2.6 m in height, and with 16-mm mesh were deployed before dusk, closed at 23:00 hours, and checked every 15 minutes. Harp traps were set from 18:00–6:00 hours and checked regularly. Bats were removed carefully from nets and harp traps and placed individually in clean cotton bags before processing. Bats were handled in accordance with guidelines that complied with Cameroonian, EU, and Nagoya Protocol regulations and which are approved by the American Society of Mammalogists (Sikes et al. 2011). Captured bats were sexed and aged, based on ossification of the finger epiphyses (Racey 1974). Standard measurements were taken, and wing tissue were collected for further genetic analysis in the laboratory. Additional samples from the tissue collections of Doñana Biological Station (EBD-CSIC), Seville, Spain were also sequenced and incorporated into the analyses, including material from mainland Equatorial Guinea, the ‘terra typica’ of *G. beatrix* (see Suppl. material 1). Bats were subsequently released close to their capture sites. However, for bats whose identification was problematic, voucher specimens were taken and preserved in 70° ethyl alcohol. Eight individuals from LNP, and two individuals from DBR were assigned to the genus *Glauconycteris* based on diagnostic morphological characters, following Happold and Happold (2013), including dentition, cranial features, and wing morphology. In addition, *G. argentata* from Mount Manengouba was also included, based on one specimen, previously reported, but without details, in Bakwo-Fils et al. (2021). These eleven specimens (AD.2023.07.18.18; AD.2023.11.19.151; AD.2023.11.25.181; AD.2023.11.25.182; AD.2023.11.28.196; AD.2024.22.05.215; AD.2024.22.05.222; AD.2024.Z73; MAN.177; Mes 26; Mes 38) are currently deposited at the Laboratory of Zoology of the University of Yaoundé I.

### Acoustic characters

Echolocation calls were obtained for specimen AD.2024.22.05.215 only. They were recorded in full spectrum (FS) file format with an EchoMeter Touch 2 PRO (Wildlife Acoustics Inc.) from hand-release bats in a flight cage. The cage was constructed from mosquito nets attached to 1.6 × 1.6 m fixed poles. Each bat was released inside the cage until its calls were successfully recorded. Calls were analysed in Kaleidoscope Pro software (v. 5.7.0, Wildlife Acoustics Inc.). Only call sequences with ≥ 3 pulses were retained for analysis. The sequences were characterised based on quantifying parameters of the call structure, namely: Fppeak (frequency which has the highest (Peak) energy within the selection), Fmax (average maximum frequency detected in the call), Fmin (average minimum frequency detected in the call), Fstart (start frequency (low) of the selection), Fend (end frequency (high) of the selection) and Dur (average duration of call pulses within the selection) (Moen et al. 2018; Fraser et al. 2020; Zamora-Gutierrez et al. 2020).

### Identification of specimens

Provisional identification of *Glauconycteris* specimens was based on diagnostic characters compared to type descriptions and summaries included in Rosevear (1965), Happold and Happold (2013), Van Cakenberghe et al. (2017), Hassanin et al. (2018), Monadjem et al. (2020a), and the African Chiroptera Report (ACR 2024). Data on previous collecting localities were based on Monadjem et al. (2024).

### External and craniodental measurements

All external measurements were taken in the field to the nearest 0.1 mm using dial callipers and subsequently checked from alcohol-preserved specimens in the laboratory. All craniodental measurements were taken to the nearest 0.01 mm with digital callipers under a stereomicroscope. Abbreviations of external and craniodental measurements (in mm) follow Bates and Harrison (1997):

**FA** forearm length

**TIB** tibia length

**HF** foot length

**E** ear length

**3MET, 4MET, 5MET** lengths of third, fourth, fifth metacarpals

**1P3D, 2P3D** lengths of first and second phalanges of the third digit

**1P5D, 2P5D** lengths of first and second phalanges of the fifth digit

**GLB** greatest length of baculum

**GSL** greatest skull length

**CCL** condylo-canine length

**ZB** zygomatic breadth

**BH** braincase height

**PC** postorbital constriction

**MB** mastoid breadth

**C–M^3^** upper toothrow length

**M^3^–M^3^** palatal width

**c–m_3_** lower toothrow length

**MAND** mandible length

Tooth abbreviations follow standard usage (**I, C, P, M** for upper teeth; **i, c, p, m** for lower teeth). The numbering of the teeth reflects homologous positions in the ancestral dentition (e.g. the anterior upper incisor is referred to as I^2^ and the second incisor as I^3^) (Miller 1907). Definitions of measurements are provided in Suppl. material 2. All measurements were from adults (as indicated by the presence of fully ossified metacarpal-phalangeal joints), except for the subadult specimen of *G. gleni* (AD.2024.22.05.222).

### Baculum preparation

After removal, by cutting close to the body, the penis was simmered for two minutes in boiling water and then soaked for 24 hours in 5% potassium hydroxide (KOH) with alizarin red stain. The baculum was manually dissected from its tissue under a microscope, measured, illustrated, and preserved in glycerine.

### Photographs

All photographs were taken with a Canon EOS R7 camera and a Sigma 70 mm F2.8 DG MACRO lens. Bacula were photographed through a microscope lens at ISO 1000 and f/4.5-f/5.6, whereas tragi, skulls, and dentition were photographed at ISO 400 and f/11- f/13. In addition to natural daylight, spot lighting was provided by two Adaptalux white lighting arms. Images were compiled in Inkscape v. 1.4.2 (https://inkscape.org/).

### DNA extraction, amplification, and sequencing

Genomic DNA was extracted from the tissue samples using a saline protocol (Gemmell and Akiyama 1996). To obtain comparable information, the same markers used by Hassanin et al. (2018), were amplified and sequenced in this study. These markers included three mitochondrial (mtDNA) fragments from the cytochrome oxidase subunit I gene (COI), the cytochrome b gene (Cytb), and the 12S rRNA gene (12S); and four nuclear (nDNA) markers from the intron 10 of the histone deacetylase 2 gene (HDAC2), the intron 6 of the RIO kinase 3 gene (RIOK3), the intron 6 of the zinc finger FYVE domain-containing 27 gene (ZFYVE27), and from the recombination activating gene 2 (RAG2). Additionally, another group of six introns, consisting of Abhydrolase domain containing 11 gene (ABHD11) intron 5, Acyl-CoA oxidase 2 (ACOX2) intron 3, Signalosome subunit 7A (COPS7A-4) intron 4, Rogdi atypical leucine zipper (ROGDI) intron 7, Actin family pseudogene 1 (ACTP1) intron 4, and Signal transducer and activator of transcription (STAT5) intron 5, were amplified for a subset of the samples.

PCR amplification of the mtDNA markers was performed using the primers: UTyrLA and C1-L705 for COI (Hassanin et al. 2012), CB-GLU-CH2 and CB-LTHR-CH for Cytb (Hassanin 2014), 12S-U1230M2-CH (Hassanin et al. 2012) and 12S-L2226M1 (Hassanin et al. 2012) for 12S. The primers used for the amplification of the nuclear introns followed Hassanin et al. (2013), while RAG2 was amplified using the primers RAG2-CHU and RAG2-CHL (Hassanin et al. 2013). Information for the primer pairs of the second set of six markers is described in Igea et al. (2010).

The PCR reaction (20 µl final volume) included 2 µl of DNA extract, 1 µl of each primer (10 µM), 0.8 µl of MgCl2 (50 mM), 0.16 µl of dNTP (25 mM), 0.2% BSA, 0.5 units of Taq Polymerase. Thermocycling consisted of 4-min initial denaturation at 94 °C followed by 35 cycles of 45 s at 94 °C, 45 s at 52 °C, and 60 s at 72 °C, and a final extension of 5 min at 72 °C. PCR products were resolved by electrophoresis on a 1.5% agarose gel stained with Syber Green and visualised under UV light.

Products were sequenced, edited, and assembled using Geneious Prime v. 2025.0.5. Heterozygous positions (double peaks) were identified and coded using IUPAC ambiguity codes. In some individuals, two alleles were sequenced in parallel and several other samples showed large insertions or deletions that required filtering the sequences with the software Gblocks 0.9.1b to eliminate possible divergent regions (Castresana 2000). All sequences generated in this study have been deposited in the GenBank database (for accession numbers and more details, see Suppl. material 1).

### Phylogenetic analyses

Sequences of four specimens (230718Gsp18, 231119Gsp151, 231125Gsp182, 240522Gbe215) from LNP were compared to homologous sequences of *Glauconycteris* obtained from the tissue collections of the EBD-CSIC, plus a GenBank selection representing nine of the 13 species known within *Glauconycteris* (Suppl. material 1) and including 34 sequences. This data set was used to prepare two alignments: 1) considering only the three mtDNA markers and 2) considering concatenated all mtDNA and nDNA sequences used in the alignment of Hassanin et al. (2018). A third alignment was prepared for a subset of samples (21 sequences) and six extra introns selected from Igea et al. 2010).

For all three alignments, the sequences were aligned using the algorithm in MUSCLE (Edgar 2004) embedded in MEGA 11 (Tamura et al. 2021). Evolutionary relationships were inspected according to Bayesian (BI) and Maximum Likelihood (ML) criteria, using *Glauconycteris superba* as an outgroup and different evolutionary models selected by jModelTest v. 0.1.10 (Darriba et al. 2012) based on the AIC criterion and run by marker. The BI phylogenetic hypotheses were obtained using MrBayes v. 3.2.1 (Ronquist et al. 2012) and after running 5.5 × 10^6^ generations with four chains, sampling every 300^th^ generation in two simultaneous runs, discarding the first 25% of trees as burn-in. The ML reconstructions were obtained in PAUP* v. 4.0b10 (Swofford 2002), using heuristic search and support estimated after 1,000 bootstrap iterations (Felsenstein 1985). Support for BI and ML was considered strong for posterior probabilities (PP) and bootstrap (BS) values ≥ 90 and 75, respectively.

Levels of genetic differentiation between species were estimated using MEGA 11 (Tamura et al. 2021) according to the Kimura 2-parameter (K2P) evolution model, since this estimate of genetic distance is conducive to comparison with other studies.

## Results

### Species identification

During four field surveys conducted in Lobéké National Park (LNP) and the Dja Biosphere Reserve (DBR), ten bats were captured that were unambiguously assigned to the genus *Glauconycteris*. All specimens exhibited the diagnostic characters of the genus as defined by Happold and Happold (2013), including characteristic dentition, ear morphology, and wing proportions (Tables 1, 2, 3). In addition, all examined males possessed a characteristically dorso-ventrally flattened penis with a broad, trilobate tip.

Of the two *Glauconycteris* specimens obtained from DBR, one was identified as *G. superba*, representing the first record of this species from Cameroon, and the other as *G. egeria*. The *Glauconycteris* specimens collected from LNP were assigned, based on morphology, to *G. beatrix*, *G. curryae*, *G. gleni*, and *G. variegata*. Three additional specimens from LNP (AD.2023.11.19.151, AD.2024.22.05.215, and AD.2023.07.18.18) could not be assigned unambiguously to any known species owing to their distinctive morphological characters. Consequently, molecular analyses of their sequences were undertaken to clarify their evolutionary relationships and assess their taxonomic status.

### Phylogenetic analyses

**Figure 2.**
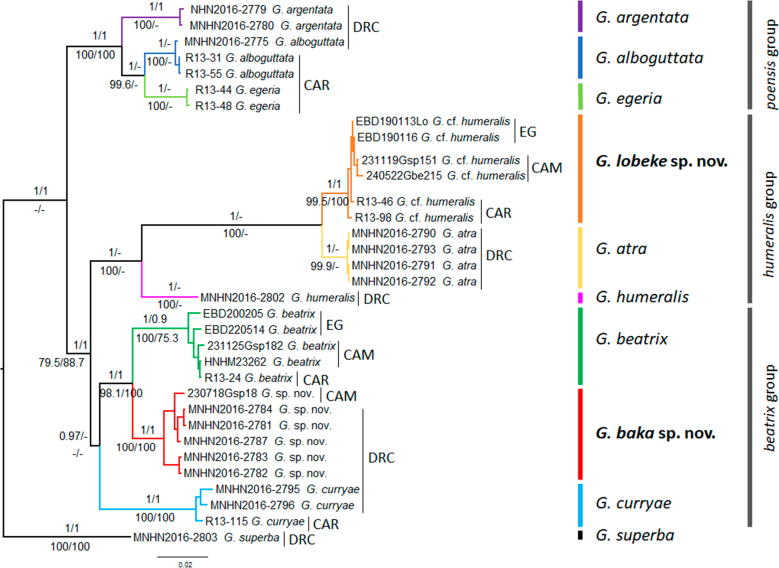
Bayesian phylogenetic reconstruction based on the concatenated mtDNA (Cytb, 12S, COI) and nuclear (HDAC2, RAG2, RIOK3, ZFYVE27) markers. Values above branches represent Bayesian posterior probabilities, and values below branches are bootstrap support in Maximum Likelihood reconstruction (support values < 75 are not shown). Values on the right (after /) correspond to the support of BI and ML obtained in the reconstructions based on a set of 6 extra introns (ABHD11, ACOX2, COPS7A-4, ROGDI, ACTP1, STAT5) that were amplified for a subset of the samples. See text for the tree's details and (Suppl. material 4) for the topology. Cameroon (CAM); Central African Republic (CAR); Democratic Republic of Congo (DRC); Equatorial Guinea (EG).

All the phylogenetic reconstructions supported similar topologies, irrespective of the dataset and analytical approach used (Fig. 2, Suppl. materials 3, 4). The mtDNA and combined mtDNA–nDNA topologies were nearly identical and broadly consistent with those presented by Hassanin et al. (2018). Our phylogeny distinguished two supported clades within the *beatrix* species group and, in addition, recovered the lineage previously referred to as *G. cf. humeralis* by Hassanin et al. (2018), which did not form a monophyletic group with *G. humeralis* in our topology (Fig. 2).

One of the two supported clades in the *beatrix* group clearly represented the species *G. beatrix* s.s. as this clade included the samples from the type locality (mainland Equatorial Guinea). This clade also included the specimen AD.2023.11.25.182 (= 231125Gsp182) from LNP. The second clade within the *beatrix* group included the specimen AD.2023.07.18.18 (= 230718Gsp18), also from LNP, that was morphologically distinct and clustered with samples from DRC reported by Hassanin et al. (2018) (Fig. 2).

Meanwhile, the sample AD.2023.11.19.151 (= 231119Gsp151) clustered together with AD.2024.22.05.215 (= 240522Gbe215) and another two from Equatorial Guinea in the group of the potential new species G. cf. humeralis of Hassanin et al. (2018) (Fig. 2).

The K2P genetic distance, based on the mtDNACytb, between *G. beatrix* s.s. and *Glauconycteris* sp. A in the *beatrix* group was 8.9%. and 4.7% between *G. cf. humeralis* and *G. atra* (Table 4).

In summary, the molecular results support the differentiation at species level of the two lineages, taxonomically unassigned due to their unique morphological features. Therefore, we proceed to their formal description as new species.

### Systematic descriptions

**Family Vespertilionidae Gray, 1821**

**Tribe Vespertilionini Gray, 1821**

**Genus *Glauconycteris* Dobson, 1875**

#### Glauconycteris baka sp. nov.

Taxon classificationAnimaliaChiropteraVespertilionidae

5DDB12AE-9F58-55DD-B94F-F7D962005C06

https://zoobank.org/4BD1ADAC-C46B-49B4-9537-58E60D97FEEB

Glauconycteris beatrix (partim): Hassanin et al. (2018: fig. 4, clades 2a and 2b, MNHN specimens from DRC).

##### Type material.

***Holotype***. Adult male (field number AD.2023.07.18.18), to be deposited at the Harrison Institute UK, for safekeeping prior to establishment of a formal collection at the Laboratory of Zoology, the University of Yaoundé I, Cameroon. Body preserved in alcohol, skull and baculum extracted, cleaned, and conserved. Collected by Aicha Gomeh-Djame, Junior Abiazhem and Eugenie Agodigo on 18 July 2023. The Cytb sequence is available in GenBank (PZ425896) (Suppl. material 1).

##### Referred material.

Material attributable to *G. baka* sp. nov. includes specimens from DRC held in the Muséum national d’Histoire naturelle (Paris), previously included within *G. beatrix* sensu Hassanin et al. (2018: fig. 4, clades 2a and 2b).

##### Type locality.

Mambele village, within the Zone of Hunting Interests with Community Management (ZICGC) of Lobéké National Park (LNP), East Region, Cameroon (2°26.63'N, 15°26.07'E), 520 m a.s.l.

##### Diagnosis.

**Figure 3.**
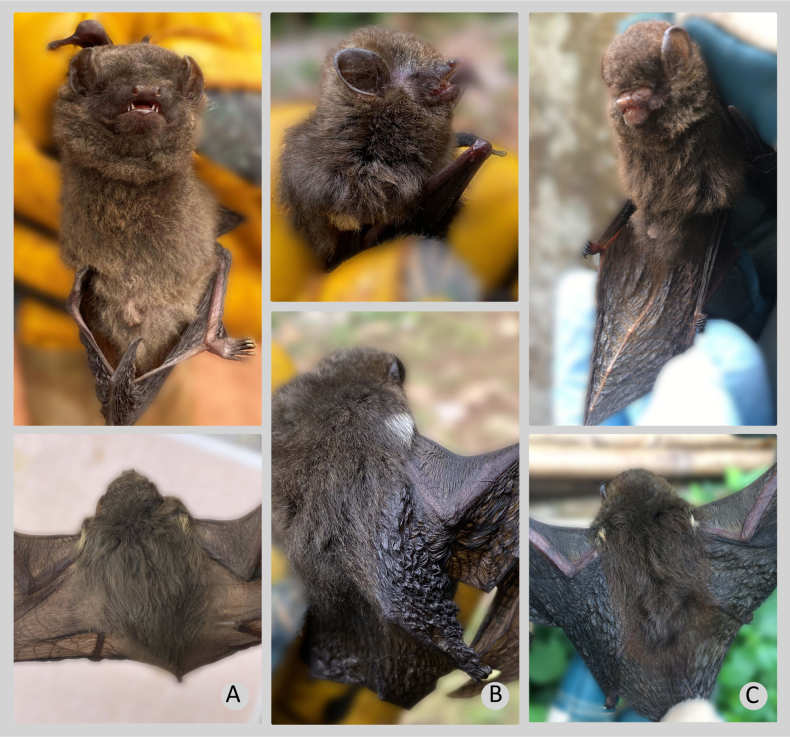
Three small, morphologically similar species of *Glauconycteris* from Cameroon with dark pelage and wings, a pale shoulder spot and no lateral stripe shown at an enlarged scale to highlight diagnostic external characters. Ventral/lateral views above, dorsal view below. **A**. *G. lobeke* sp. nov., ♂, holotype, AD.2023.11.19.151, Djombi village, LNP; **B**. *G. beatrix*, ♂, AD.2023.11.25.182, Pont cassé platform, LNP; **C**. *G. baka* sp. nov., ♂, holotype, AD.2023.07.18.18, Mambele village, LNP. See Fig. 8 for additional species of the genus. Not to scale.

**Figure 4.**
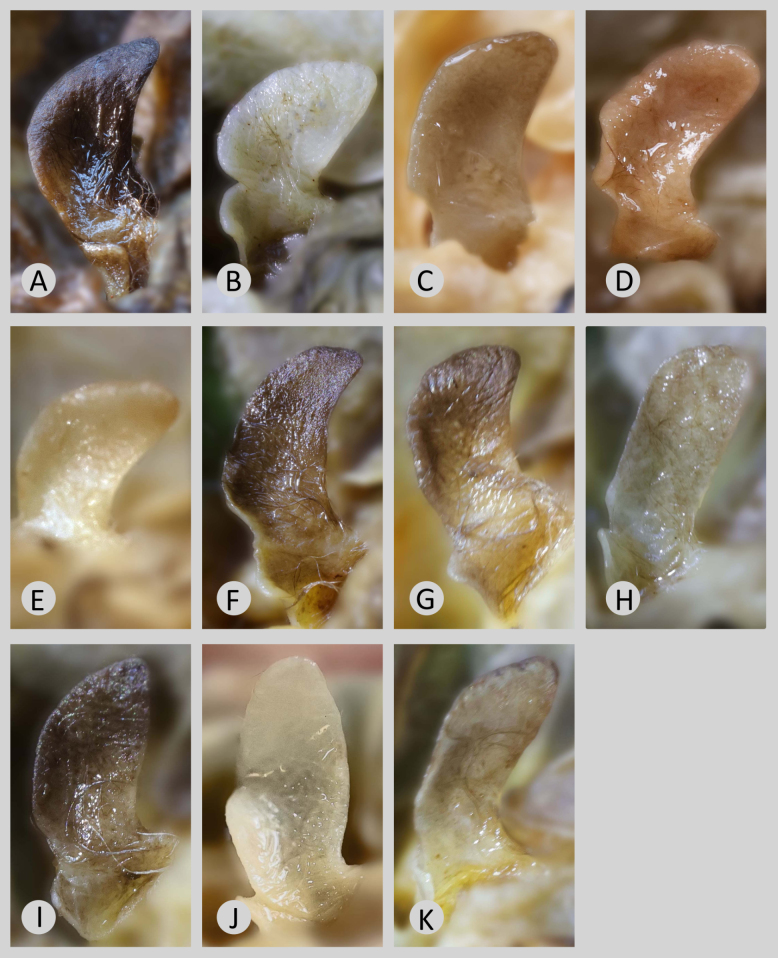
Tragi of ten of the 11 species of *Glauconycteris* known from Cameroon, with *G. humeralis* for comparative purposes. Species order follows Table 1. **A**. *G. superba*, ♂, Mes 26, Mba’a village, DBR; **B**. *G. gleni*, ♂ (subadult), AD.2024.22.05.222, Yenga fishing site, LNP; **C**. *G. variegata*, ♀, HZM.7.39901, Barotseland, Zambia; **D**. *G. alboguttata*, ♀, HZM.1.40172, Lekoumou, Congo; **E**. *G. argentata*, ♂, Mbouroukou école publique, Mount Manengouba National Park; **F**. *G. egeria*, ♀, Blandjock village, DBR; **G**. *G. lobeke* sp. nov., ♂, holotype, AD.2023.11.19.151, Djombi village, LNP; **H**. *G. curryae*, ♀, AD.2023.11.28.196, Bai petite savane platform, LNP; **I**. *G. beatrix*, ♂, AD.2023.11.25.182, Pont cassé platform, LNP; **J**. *G. humeralis*, ♂, HZM.1.8023, Avakuhi, DRC; **K**. *G. baka* sp. nov., ♂, holotype, AD.2023.07.18.18, Mambele village, LNP. All specimens from Cameroon, unless otherwise stated. Not to scale.

**Figure 5.**
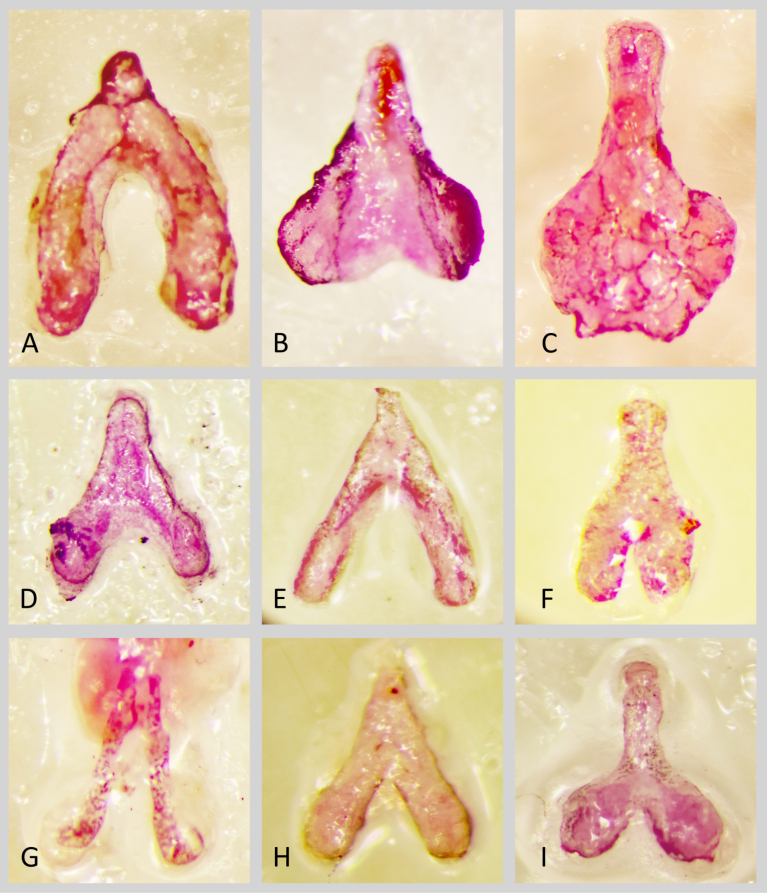
Bacula of eight of the 11 species of *Glauconycteris* known from Cameroon, with *G. humeralis* for comparative purposes. Species order follows Table 1. **A**. *G. superba*, Mes 26, Mba’a village, DBR; **B**. *G. gleni*, (subadult), AD.2024.22.05.222, Yenga fishing site, LNP; **C**. *G. variegata*, HZM.1.1770, Garissa, Kenya; **D**. *G. argentata*, Mbouroukou école publique, Mount Manengouba National Park; **E**. *G. lobeke* sp. nov., holotype, AD.2023.11.19.151, Djombi village, LNP; **F**. *G. curryae*, AD.2023.11.25.181, Pont cassé platform, LNP; **G**. *G. beatrix*, AD.2023.11.25.182, Pont cassé platform, LNP; **H**. *G. humeralis*, HZM.1.8023, Avakuhi, DRC, **I**. *G. baka* sp. nov., holotype, AD.2023.07.18.18, Mambele village, LNP. Specimens from Cameroon, unless otherwise stated. Not to scale.

**Figure 6.**
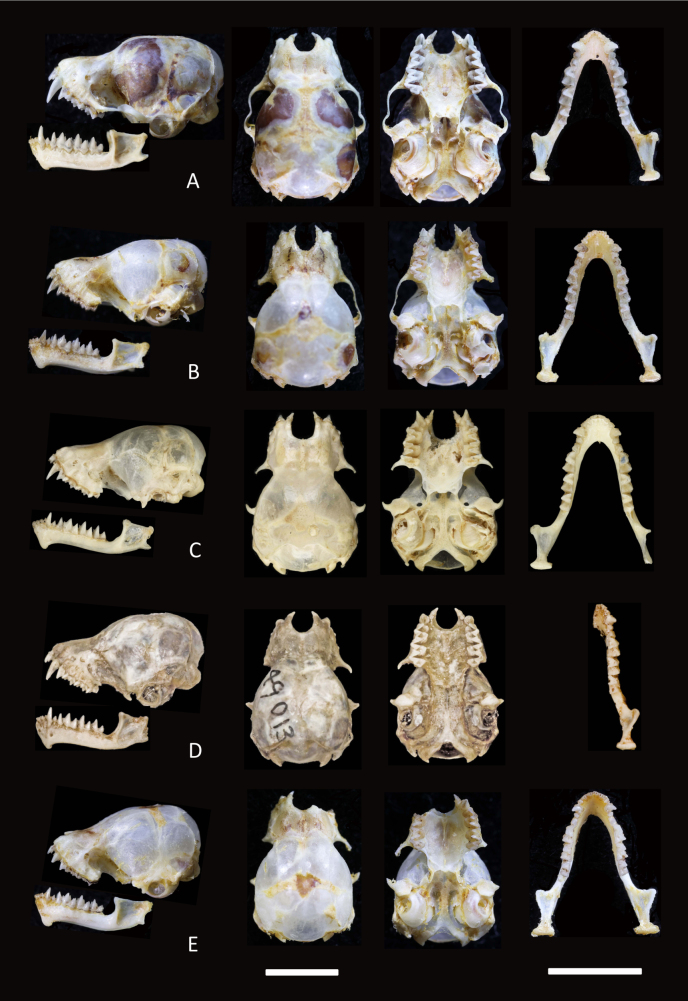
Skulls of the three species of small *Glauconycteris* from Cameroon with dark pelage and wings, a pale shoulder spot and no lateral stripe, with *G. humeralis* for comparative purposes. **A**. *G. lobeke* sp. nov., ♂, holotype, AD.2023.11.19.151, Djombi village, LNP; **B**. *G. beatrix*, ♂, AD.2023.11.25.182, Pont cassé platform, LNP; **C**. *G. beatrix*, ♀, holotype, NHMUK.95.5.4.19, Equatorial Guinea; **D**. *G. humeralis*, ♀, holotype, AMNH M49013, Medje, DRC [lateral view is reversed]; **E**. *G. baka* sp. nov., ♂, holotype, AD.2023.07.18.18, Mambele village, LNP. Specimens from Cameroon, unless otherwise stated. Scale bars: 5 mm.

**Figure 7.**
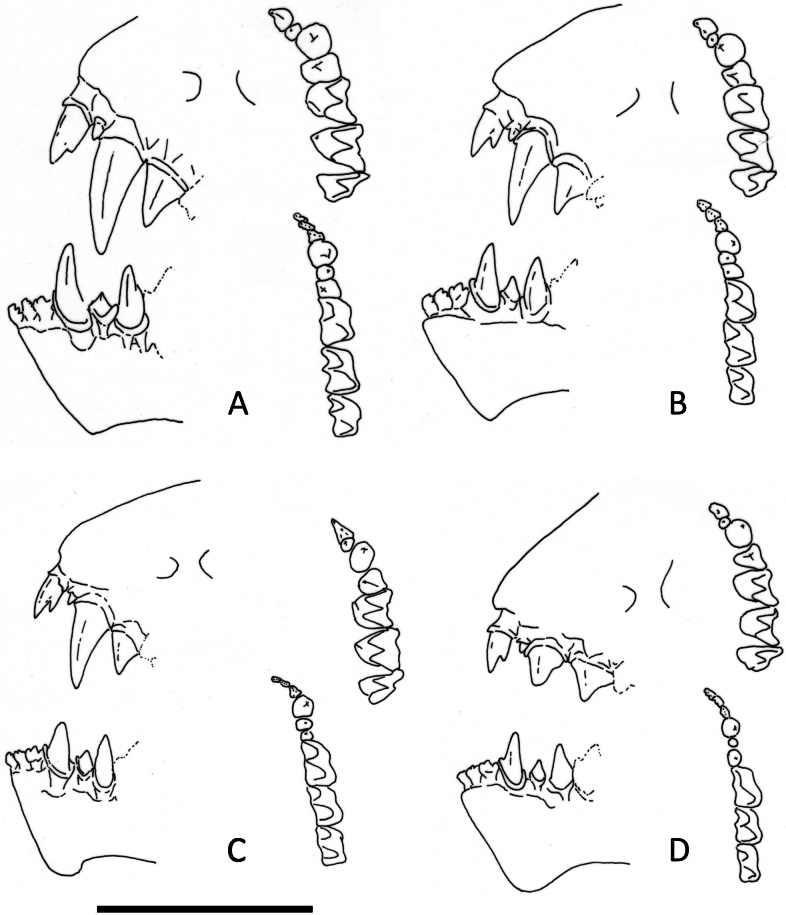
Maxillary and mandibular dentition of the three species of small *Glauconycteris* from Cameroon with dark pelage and wings, a pale shoulder and no lateral stripe, with *G. humeralis* for comparative purposes. **A**. *G. lobeke* sp. nov., ♂, holotype, AD.2023.11.19.151, Djombi village, LNP; **B**. *G. beatrix*, ♂, AD.2023.11.25.182, Pont cassé platform, LNP; **C**. *G. humeralis*, ♂, HZM.1.8023, Avakuhi, DRC; **D**. *G. baka* sp. nov., ♂, holotype, AD.2023.07.18.18, Mambele village, LNP. Specimens from Cameroon, unless otherwise stated. Scale bar: 5 mm.

A small representative of the genus (FA: 34.2 mm; GSL: 10.73 mm) (Tables 1, 2, 3). The dorsal and ventral pelage is dark brown with a small, cream-coloured spot on each shoulder; the wings and tail membrane are brown, almost black and without conspicuous reticulation (Fig. 3C). The muzzle is flesh-coloured and hairless, and the ears are rounded (Fig. 3C). The tragus is relatively narrow with a straight anterior margin and a posterior margin that is gently convex, with a rounded tip; there is a well-developed basal lobe, which is not clearly visible in Fig. 4K. The baculum is small (GLB: 0.74 mm) and shaped like an inverted capital Y, with a narrow distal shaft and expanded basal lobes (Fig. 5I, Table 2). The skull is small, with a bulbous braincase, which is noticeably expanded anteriorly (Fig. 6E). The rostrum is short and the dorsal profile is slightly concave. The second cusp of the bifid anterior upper incisor (I^2^) is well defined and is half the height of the principal cusp (Fig. 7D). The dentition is relatively weak, with a short lower canine and small lower premolars (p_2_ and p_4_) (Fig. 7D).

**Table 2.** External measurements (mm) and proportions (%) of *Glauconycteris* collected for this study, together with the holotype of *G. beatrix* and two additional comparative specimens of *G. beatrix* and *G. humeralis*. Species order follows Table 1. Measurements are defined in Materials and methods.

Species, sex, ID	FA	TIB	HF	E	5 Met	1P5D	2P5D	4 Met	3 Met	1P3D	2P3D	2P3D/1P3D × 100 (%)	GLB
*G. superba* ♂ Mes 26	46.0	18.6	10.2	13.6	38.2	8.5	7.6	42.1	42.6	20.3	25.2	124.0	1.11
*G. gleni* ♂ AD.2024.22.05.222	41.9	14.9	7.3	12.2	37.0	8.3	8.4	38.6	41.6	16.0	24.0	150.4	0.89
*G. variegata* ♂ AD.2024.Z73	41.2	19.1	6.8	13.8	38.1	7.8	8.7	36.1	36.9	15.3	20.6	134.6	1.51
*G. argentata* ♂ MAN.177	42.2	18.2	5.0	11.0	36.2	9.4	7.3	40.5	43.1	14.6	27.0	185.4	0.87
*G. egeria* ♀ Mes 38	40.0	17.4	8.5	11.9	35.6	7.9	8.4	37.3	39.6	14.7	27.2	184.6	×
*G. curryae* ♂ AD.2023.11.25.181	35.1	14.8	6.3	9.4	31.3	8.7	6.9	33.4	35.8	14.6	21.1	144.5	0.55
*G. curryae* ♀ AD.2023.11.28.196	37.9	15.7	5.5	12.3	33.1	8.9	7.7	35.8	37.6	14.0	19.0	135.7	×
*G. lobeke* sp. nov. (holotype) ♂ AD.2023.11.19.151	35.8	17.5	7.2	7.5	31.5	8.0	7.0	33.8	34.0	12.3	22.1	179.7	0.66
*G. lobeke* sp.nov. (paratype) ♀ AD.2024.22.05.215	35.9	14.8	6.4	9.8	31.5	8.5	6.7	34.4	36.1	12.6	21.2	168.3	×
*G. beatrix* ♂ AD.2023.11.25.182	36.3	18.4	6.0	9.2	30.8	8.6	8.2	33.5	37.0	12.1	21.6	178.5	0.57
*G. beatrix* (holotype) ♀ NHMUK 96.5.4.19	39.2	19.2	6.4	×	35.6	8.9	8.3	36.9	39.7	13.2	23.8	180.1	×
*G. beatrix* ♀ HZM.1.40181	41.0	21.4	6.6	×	37.6	9.8	8.5	40.0	43.1	13.9	24.7	177.6	×
*G. humeralis* ♂ HZM.1.8023	36.6	18.7	7.0	×	33.4	9.8	7.1	35.5	36.7	13.9	19.6	141.1	0.60
*G. baka* sp.nov. (holotype) ♂ AD.2023.07.18.18	34.2	15.6	5.4	10.1	30.8	9.3	8.5	32.9	35.5	12.1	22.4	185.1	0.74

##### Etymology.

The species is named in honour of the Baka people, who live in and around LNP, and who have a close relationship with the forest and its wildlife. The name was discussed with members of the local community, including through park rangers, and was positively received, with community members expressing pride in the association. The proposed French name is ‘Chauve-souris papillon Baka’ and the proposed English name is ‘Baka butterfly bat’.

##### Description.

This is a small *Glauconycteris* with a FA of 34.2 mm and a TIB of 15.6 mm (Table 2). The dorsal and ventral pelage are dark brown; the hairs are bicoloured, blackish brown at the base and brownish at the tip; there is a small cream-coloured spot on each shoulder but no dorsolateral flank stripe (Fig. 3C). The pelage extends onto the inner margins of the interfemoral membrane and plagiopatagium. The muzzle is flesh-coloured and hairless. The wings and the interfemoral membrane are almost black and without conspicuous reticulation. In the wing, the second phalanx of the third digit (2P3D) is long compared to the first phalanx and compared to FA; the phalanges of the fifth digit are long in comparison the fifth metacarpal (5MET) (Table 2). The ears are brown, separated, distinctly rounded, and 10.1 mm in height. Each tragus is comparable in colour to the ear and is relatively narrow, with a straight anterior margin and a posterior margin that is gently convex, with a rounded tip (Fig. 4K). The penis is dorsoventrally flattened, with a broad, trilobate distal portion. The baculum is small (GLB: 0.74 mm), and shaped like an inverted capital Y, with a straight, narrow distal portion and two bulbous posterior lobes (Fig. 5I, Table 2).

The skull is small (GSL: 10.73 mm). The braincase is inflated, including its anterior part, such that postorbital constriction (PC) is 40.1% of the greatest length of skull (GSL); BH is 47.8% of GSL (Table 3). The rostrum is short, and the dorsal profile has a slight concavity (Fig. 6E). The secondary cusp of the bifid anterior upper incisor (I^2^) is well-defined and approximately half the height of the first; the posterior incisor (I^3^) is small, compressed between I^2^ and the canine and lies within the toothrow; the length of the upper canine is not known as it is reduced by wear in the holotype (Fig. 7D). The upper premolar and three molars are unremarkable. The three lower incisors are compressed and well defined; the lower canine has a small crown area and is short; the lower premolars (p_2_ and p_4_) are reduced; the three lower molars are unremarkable (Fig. 7D).

**Table 3:** Cranial measurements (mm) and proportions (%) of *Glauconycteris* collected for this study, together with the holotype of *G. beatrix* and two additional comparative specimens of *G. beatrix* and *G. humeralis*. Species order follows Table 1. Measurements are listed in Materials and methods.

Species, sex, ID	GSL	CCL	ZB	BB	PC	PC/GSL × 100 (%)	BH	BH/GSL × 100 (%)	MB	C-M^3^	M^3-^M^3^	c-m_3_	MAND
*G. superba* ♂ Mes 26	16.40	15.41	11.40	8.63	4.78	29.1	7.14	43.5	9.53	5.64	7.80	6.30	12.05
*G. gleni* ♂ AD.2024.22.05.222	13.84	13.27	9.43	7.78	4.50	32.5	5.71	41.3	8.61	4.67	5.89	5.05	9.85
*G. argentata* ♂ MAN.177	12.27	11.59	8.94	7.27	5.04	41.1	5.88	47.9	8.06	4.10	5.94	4.62	8.89
*G. egeria* ♀ Mes 38	13.72	13.04	9.05	7.64	3.99	29.1	5.50	40.1	8.12	4.36	5.87	4.74	9.42
*G. curryae* ♂ AD.2023.11.25.181	×	11.11	8.51	6.84	4.00	×	4.95	×	7.28	4.13	5.51	4.40	8.34
*G. curryae* ♀ AD.2023.11.28.196	12.41	11.49	8.92	7.07	4.25	34.2	5.24	42.2	7.96	4.21	5.83	4.61	8.58
*G. lobeke* sp. nov. (holotype) ♂ AD.2023.11.19.151	12.24	11.33	8.39	6.64	4.41	36.0	5.51	45.0	7.48	4.19	5.55	4.56	8.41
*G. lobeke* sp.nov. (paratype) ♀ AD.2024.22.05.215	12.34	11.63	8.87	6.93	4.33	35.1	5.41	43.8	7.73	4.29	5.73	4.61	8.86
*G. beatrix* ♂ AD.2023.11.25.182	11.56	10.99	×	6.81	3.99	34.5	4.98	43.1	7.45	3.83	5.21	4.17	8.40
*G. beatrix* (holotype) ♀ NHMUK96.5.4.19	11.38	11.05	×	7.08	4.29	37.7	5.11	44.9	7.47	4.13	5.71	4.55	8.82
*G. beatrix* ♀ HZM.1.40181	11.77	11.23	8.51	7.12	4.12	35.0	5.44	46.2	7.62	4.04	5.79	4.48	8.51
*G. humeralis* ♂ HZM.1.8023	11.18	10.59	8.25	7.09	4.19	37.5	5.20	46.5	7.45	4.05	5.42	4.20	8.01
*G. baka* sp.nov. (holotype) ♂ AD.2023.07.18.18	10.73	10.43	×	6.77	4.30	40.1	5.13	47.8	7.28	3.72	5.02	4.09	8.28

No echolocation calls were obtained for this species, and no acoustic data are therefore available.

##### Comparison with similar species.

**Figure 8.**
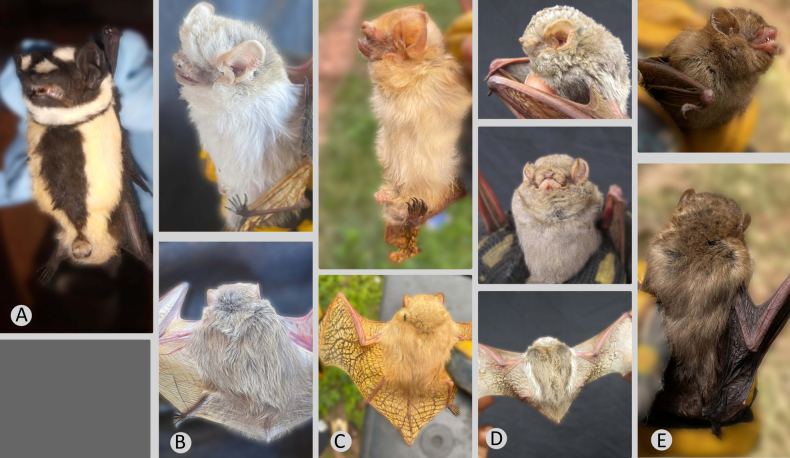
Additional species of *Glauconycteris* recorded in Cameroon, illustrating broader variation in size and pelage across the genus. Species order follows Table 1. **A**. *G. superba*, ♂, Mes 26, Mba’a village, DBR; **B**. *G. gleni*, ♂, (subadult), AD.2024.22.05.222, Yenga fishing site, LNP; **C**. *G. variegata*, ♂, AD.2024.Z73, Zega village, LNP; **D**. *G. argentata*, ♂, Mbouroukou école publique, Mount Manengouba National Park; **E**. *G. curryae*, ♂, AD.2023.11.25.181, Pont cassé platform, LNP. The focal, closely related species are illustrated at larger scale in Fig. 3. Not to scale.

**Figure 9.**
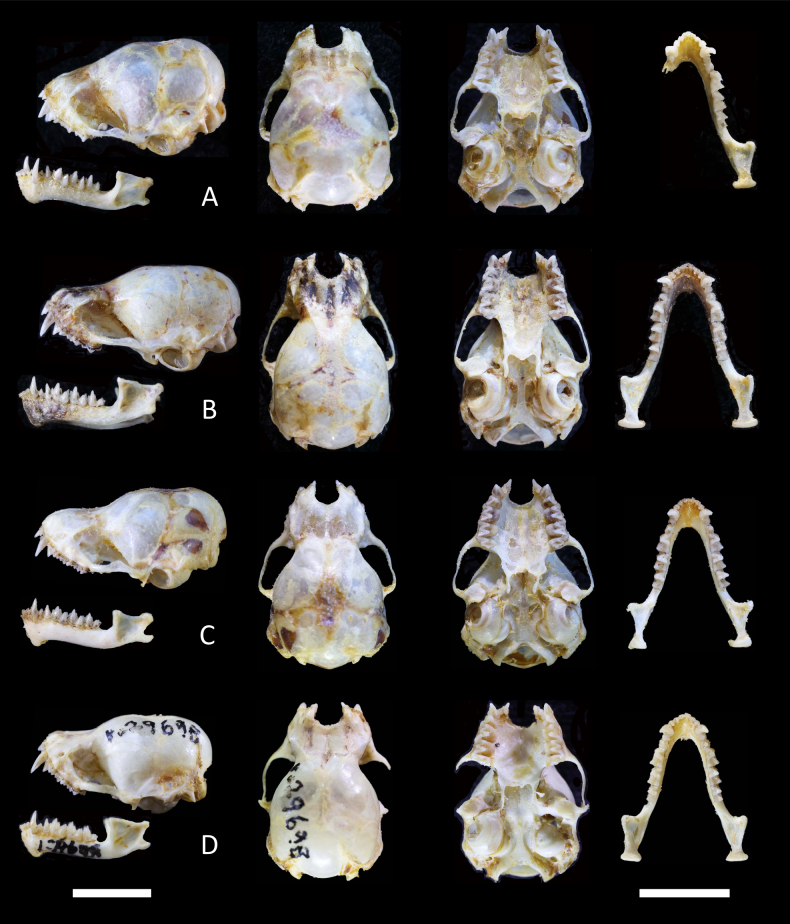
Skulls of medium sized *Glauconycteris* known from Cameroon. Species order follows Table 1. **A**. *G. argentata*, ♂, Mbouroukou école publique, Mount Manengouba National Park; **B**. *G. egeria*, ♀, Blandjock village, DBR; **C**. *G. curryae*, ♀, AD.2023.11.28.196, Bai petite savane platform, LNP; **D**. *G. poensis*, sex unknown, HZM.1.29698, Bonthe District, Sierra Leone. Specimens from Cameroon, unless otherwise stated. Scale bars: 5 mm.

**Figure 10.**
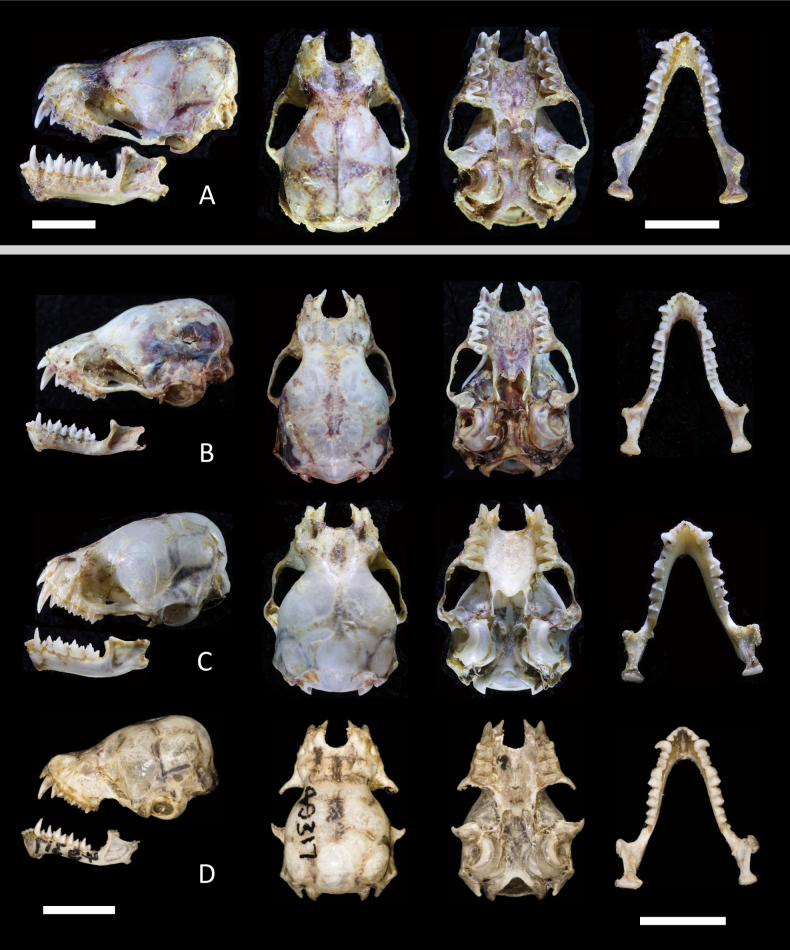
Skulls of larger sized *Glauconycteris* known from Cameroon. Species order follows Table 1. **A**. *G. superba*, ♂, Mes 26, Mba’a village, DBR; **B**. *G. gleni*, ♂ (subadult), AD.2024.22.05.222, Yenga fishing site, LNP; **C**. *G. variegata*, ♀, HZM.7.39901, Barotseland, Zambia; **D**. *G. alboguttata*, ♀, holotype, AMNH M49317, Medje, DRC [lateral view is reversed]. Specimens from Cameroon, unless otherwise stated. Scale bars: 5 mm.

*Glauconycteris baka* sp. nov. clearly differs from *G. superba*, *G. gleni*, *G. variegata*, *G. machadoi*, *G. alboguttata*, *G. argentata*, *G. kenyacola*, and *G. egeria*, by its smaller size (Tables 1, 2, 3) combined with a variety of other characters, including pelage colour and absence of wing reticulation (Figs 3, 8), shape of tragus (Fig. 4), bacular morphology (Fig. 5), cranial and dental morphology (Figs 6, 9, 10), and genetics (Fig. 2, Table 4, Suppl. materials 3, 4).

**Table 4.** Genetic distances (Kimura 2-parameter [K2P] distances) based on the partial mitochondrial Cytb gene among the species studied within the genus *Glauconycteris*. (*Glauconycteris* sp. A = *G. baka* sp. nov. and *G. cf. humeralis* = *G. lobeke* sp. nov. when comparing with provisional identifications used in the Results).

	*G. lobeke* sp. nov.	*G. beatrix* s.s.	*G. baka* sp. nov.	*G. argentata*	*G. humeralis* s.s.	*G. atra*	*G. curryae*	*G. alboguttata*	*G. egeria*	*G. superba*
*G. lobeke* sp. nov.										
*G. beatrix* s.s.	0.166									
*G. baka* sp. nov.	0.148	0.089								
*G. argentata*	0.172	0.171	0.156							
*G. humeralis* s.s.	0.135	0.176	0.157	0.195						
*G. atra*	0.047	0.157	0.138	0.172	0.132					
*G. curryae*	0.189	0.152	0.142	0.184	0.187	0.180				
*G. alboguttata*	0.191	0.172	0.150	0.119	0.181	0.187	0.174			
*G. egeria*	0.199	0.183	0.157	0.130	0.195	0.200	0.170	0.066		
*G. superba*	0.200	0.178	0.191	0.221	0.241	0.216	0.178	0.196	0.209	

Based on size, it is more similar to five *Glauconycteris* species: *G. atra*, *G. curryae*, *G. beatrix*, *G. humeralis*, and *G. poensis* (Tables 1, 2, 3). It differs from *G. poensis* in having dark brown rather than greyish brown pelage; absence of flank stripe (Hassanin et al. 2018: fig. 6); essentially straight tragus rather than more crescent-shaped (Happold and Happold 2013: fig. 124e); a Y-shaped baculum with expanded basal lobes as compared to V-shaped without expanded lobes (Happold and Happold 2013: fig. 128e, f); a small skull with reduced rostrum and slightly concave profile as compared to a skull with a strongly concave profile (Figs 6E, 9D); and genetics (Hassanin et al. 2018: fig. 4). It differs from *G. beatrix*, its closest phylogenetic species, in its Y-shaped baculum as opposed to an inverted V-shaped baculum with long, slender basal arms (Fig. 5G, I) and its reduced rostrum coupled with the noticeably inflated anterior part of the braincase (Fig. 6B, C, E). In addition to these morphological differences, the genetic distance between *G. baka* sp. nov. and *G. beatrix* is 8.9% (Table 4), a value that clearly supports the taxonomic distinction between both lineages (Fig. 2, Suppl. materials 3, 4). It differs from *G. humeralis* in its shortened rostrum (Fig. 6D, E), the shape of the baculum (Fig. 5H, I), and genetics (Fig. 2, Table 4, Suppl. material 3). In *G. humeralis*, the baculum is considered peg-shaped, based on a specimen from Uganda (Happold and Happold 2013: fig. 128d). However, in a further specimen from DRC (HZM.1.8023), which compares more favourably in cranial and dental morphology to the holotype, and which was collected some 150 km from the type locality, the baculum (GLB: 0.60 mm) is shaped like an inverted ‘V’, with basal lobes defined (Fig. 5H). It differs from *G. curryae* in bacular morphology (Fig. 5F, I) and in the shape of the skull, especially the dorsal profile of the rostrum and the braincase, which is much more globose in *G. baka* sp. nov. (Figs 6E, 9C). It is also distinct genetically (Fig. 2, Table 4, Suppl. materials 3, 4). It differs from *G. atra* in its more globose braincase, shortened rostrum (Fig. 6E; Hassanin et al. 2018: fig. 2) and genetics (Fig. 2, Table 4).

##### Distribution and ecology.

**Figure 11.**
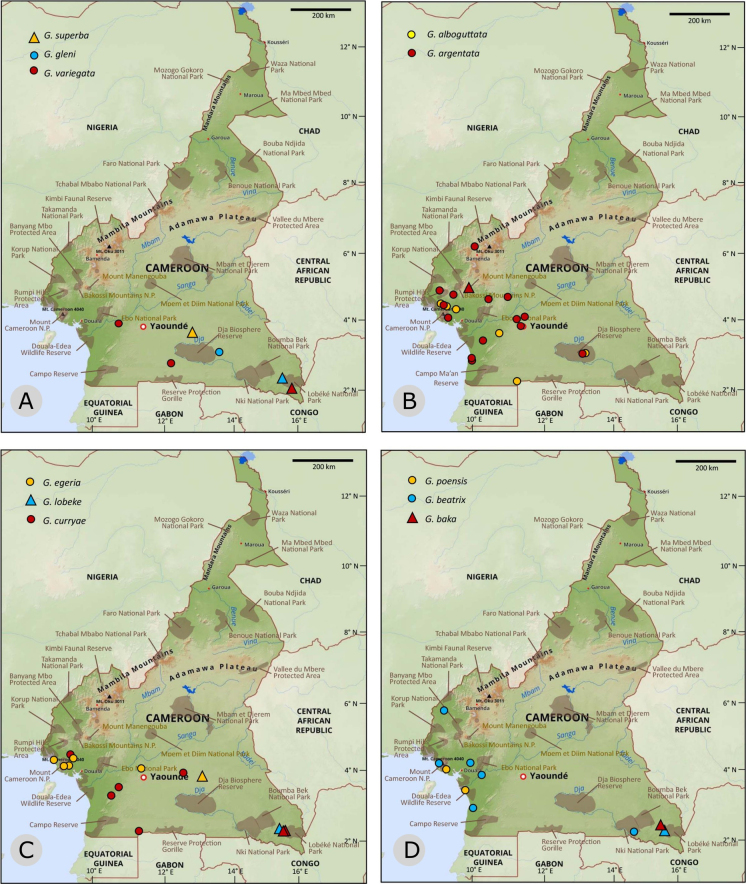
Distribution of the 11 species of *Glauconycteris* known from Cameroon. Species order follows Table 1. Triangles signify new data records; circles are for previous records published in Monadjem et al. (2024). Base map downloaded from https://www.freeworldmaps.net/africa/cameroon/map.html.

*Glauconycteris baka* sp. nov. is currently known from the type locality, near Mambele village, at an elevation of 520 m a.s.l. in the Zone of Hunting Interests with Community Management (ZICGC) in the buffer zone of LNP, Cameroon (Fig. 11D), where it was collected in a mist net set near a waterfall in a pocket of open canopy evergreen Congolian lowland forest. In addition, based on phylogenetic evidence, it is also present at Bongandjola, Melume, and Yoko (all in DRC) (Fig. 2; Hassanin et al. 2018: appendix 1).

#### Glauconycteris lobeke sp. nov.

Taxon classificationAnimaliaChiropteraVespertilionidae

C5AF2404-F388-5C65-BBB5-78DA25C08937

https://zoobank.org/43CF7E6A-8E7D-4075-B2FC-504A2911D64E

Glauconycteris cf. humeralis : Hassanin et al. (2018: fig. 4; tissue samples R13-46 and R13-98 from CAR).
Glauconycteris cf. humeralis : Torrent et al. (2025a: 22; tissue samples EBD190113Lo and EBD190116 from Equatorial Guinea).

##### Type material.

***Holotype***. Adult male (field number AD.2023.11.19.151), to be deposited at the Harrison Institute UK for safekeeping prior to establishing a formal collection at the Laboratory of Zoology, the University of Yaoundé I, Cameroon. Body preserved in alcohol, skull and baculum extracted, cleaned, and conserved. Collected by Aicha Gomeh-Djame, Junior Abiazhem and Eugenie Agodigo on 19 November 2023. The Cytb sequence is available in GenBank (PZ425897) (Suppl. material 1). ***Paratype***. Adult female (field number AD.2024.22.05.215), deposited at the Laboratory of Zoology of the University of Yaoundé I. Body preserved in alcohol, skull extracted. Collected in Yenga fishing site, Forest Management Unit (FMU) of Lobéké National Park (LNP), East Region, Cameroon (2°17.59'N, 15°21.37'E) at 560 m a.s.l. by Aicha Gomeh-Djame and Guy Marcelin Bidias Nango on the 22 May 2024. The Cytb sequence is available in GenBank (PZ425898) (Suppl. material 1).

##### Type locality.

**Figure 12.**
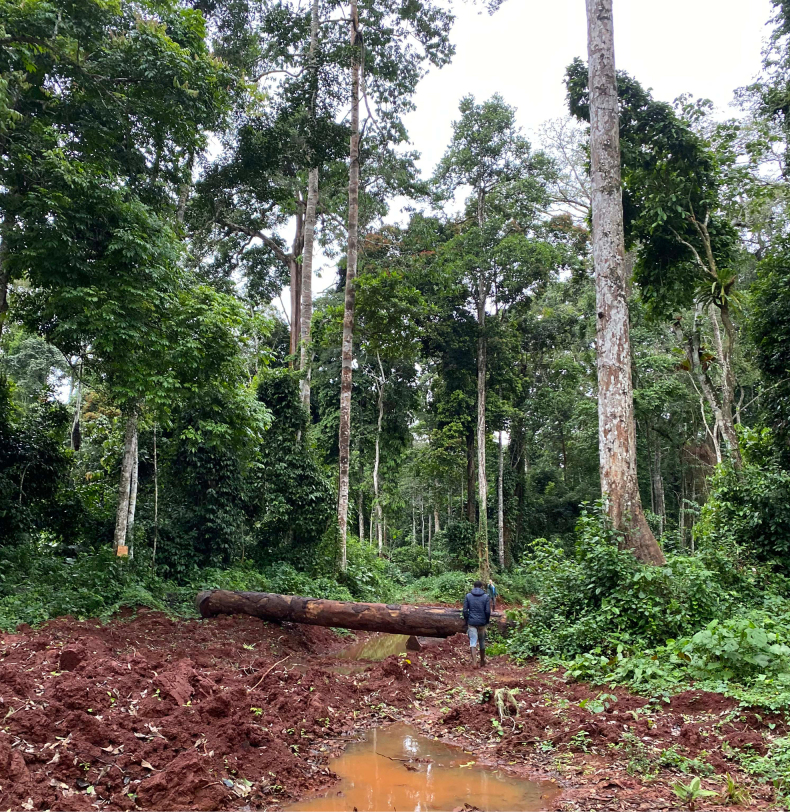
The type locality of *G. lobeke* sp. nov., situated within a Forest Management Unit of the buffer zone of LNP, characterised by small patches of heavily degraded swamp evergreen forest.

Djombi village within the Forest Management Unit (FMU) of the buffer zone of Lobéké National Park (LNP), East Region, Cameroon (2°23.70'N, 15°31.79'E), 570 m a.s.l. (Figs 11C, 12).

##### Referred material.

Material attributable to *G. lobeke* sp. nov. includes tissue samples of two individuals, R13-46 and R13-98, captured and subsequently released in CAR in 2013, and two individuals (EBD190113Lo and EBD190116), captured and subsequently released in mainland Equatorial Guinea in 2019 and 2023. All were referred to *G. cf. humeralis* based on their sequences (Hassanin et al. 2018; Torrent et al. 2025a). Potentially, four individuals, one male (TTU 150384) and three females (TTU 150405, IT2017-153 and IT2017-133), which were collected in Cross River State, Nigeria, and were referred to *G. cf. humeralis* based on their brown to blackish brown pelage colour, with characteristic white shoulder spots (Tanshi et al. 2022), could also belong to this species.

##### Diagnosis.

This is a medium-small *Glauconycteris* (FA: 35.8, 35.9 mm; GSL: 12.24, 12.34 mm) (Tables 1, 2, 3). The dorsal and ventral pelage is sepia-brown to darker brown with a pale spot on each shoulder; the wings are mid-brown and without conspicuous reticulation (Fig. 3A). The muzzle is brown, with short hairs. The ears are rounded; the tragus has a concave anterior margin, and a convex posterior margin; there is a well-defined basal lobe (Fig. 4G). The baculum is small (GLB: 0.66 mm) and shaped like an inverted capital ‘V’ with a very short distal tip and two long straight, narrow basal lobes (Fig. 5E). The skull has a bulbous braincase, especially anteriorly, and a strongly concave dorsal profile; the rostrum is broad and flattened (Fig. 6A). The secondary cusp of the bifid anterior upper incisor (I^2^) is present but not well-defined; the upper and lower canines are robust; and the lower premolars (p_2_ and p_4_) are not reduced (Fig. 7A).

##### Etymology.

The species is named after Lobéké National Park, where the individuals were captured. We wish to draw attention to the important role the park can play in protecting a highly diverse fauna of Congolian forest bats, including new and rare species. The proposed French name is ‘Chauve-souris papillon de Lobéké’ and the proposed English name is ‘Lobéké butterfly bat’.

##### Description.

This is a medium-small *Glauconycteris* with a relatively large skull (GSL: 12.24, 12.34 mm) in comparison to its FA of 35.8, 35.9 mm (Tables 1, 2, 3). The dorsal and ventral pelage is sepia-brown to darker brown. The holotype has unicoloured hairs; they are bicoloured in the paratype, basally brown but with the tips a paler, more sepia mid-brown. Both specimens have a pale spot on each shoulder; neither specimen has a dorsolateral flank stripe. The muzzle is brown, with short hair. The wings and the interfemoral membrane are mid-brown without conspicuous reticulation. The interfemoral membrane is hirsute, especially adjacent to the tail, with the hairs extending beyond the posterior margin of the membrane (Fig. 3A). The ears are brown, separated, rounded, and 7.5–9.8 mm in height. Each tragus is comparable in colour to the ear and is crescent-shaped, with a concave anterior margin and a convex posterior margin (Fig. 4G). The penis is flattened, with a broad, trilobate distal portion. The baculum is shaped like an inverted capital ‘V’, with a very short distal tip, and long, straight, narrow basal lobes (Fig. 5E). The skull is characterised by its bulbous braincase. The braincase rises steeply above the rostrum, giving a strongly concave profile; in contrast, the rostrum appears flattened (Fig. 6A). The first upper incisor (I^2^) is well developed but with a small, inconspicuous secondary cusp (Fig. 7A). The upper canine is robust and long. The upper premolar (P^4^) is also relatively robust. The three upper molars are unremarkable. The three lower incisors are compressed. The lower canine is well developed, and the lower premolars (p_2_ and p_4_) are not conspicuously reduced (Fig. 7A). The three lower molars are unremarkable.

##### Echolocation.

**Figure 13.**
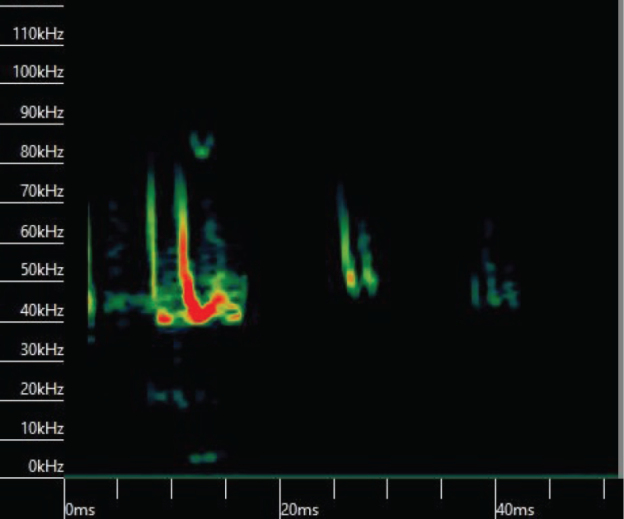
Release calls of the paratype of *G. lobeke* sp. nov. ♀, AD.2024.22.05.215, taken in a flight cage - Fstart of 37.82 kHz, Fppeak of 41.15 kHz, and Dur of 2.69 ms.

The echolocation calls of *Glauconycteris lobeke* sp. nov. are only known from the paratype. The sonotype structure is FM-QCF (Frequency Modulated-Quasi Constant Frequency) (Fig. 13) with Fppeak 41.15 kHz, Fmax 71.66 kHz, Fmin 43.97 kHz, Fstart 37.82 kHz, Fend 104.52 kHz, and Dur 2.69 ms.

##### Comparison with similar species.

*Glauconycteris lobeke* sp. nov. differs from *G. superba*, *G. gleni*, *G. variegata*, *G. machadoi*, *G. alboguttata*, *G. argentata*, *G. kenyacola*, and *G. egeria* by its smaller size (Tables 1, 2, 3) combined with a range of other characters, including pelage colour and absence of wing reticulation (Figs 3, 8), tragi morphology (Fig. 4), bacular morphology (Fig. 5), cranial and dental morphology (Figs 6, 9, 10), and genetics (Fig. 2, Table 4, Suppl. materials 3, 4).

Based on size, it is more similar to six *Glauconycteris* species: *G. atra*, *G. curryae*, *G. poensis*, *G. beatrix*, *G. humeralis*, and *G. baka* sp. nov. In comparison to *G. curryae*, which has tricoloured hairs and no shoulder spot, *G. lobeke* sp. nov. has a shoulder spot with unicoloured or bicoloured hairs (Figs 3A, 8E). The shape of baculum also differs (Fig. 5E, F), and in the skull, both have concave dorsal profiles but the braincase of *G. lobeke* sp. nov. is more developed anteriorly (Figs 6A, 9C). It differs from *G. atra* in the presence of a shoulder spot (although it is not known if this is a constant character) and genetics (Fig. 2, Table 4). It differs from *G. poensis* in having chestnut to darker brown pelage rather than greyish brown pelage and is without a dorsolateral flank stripe (Fig. 3A, Hassanin et al. 2018: fig. 6). It differs from *G. beatrix* in the shape of the skull, especially the rostrum, giving a strongly concave dorsal profile as compared to a weakly concave profile (Fig. 6A, B, C); the shape of the baculum (Fig. 5E, G); and genetics (Fig. 2, Table 4, Suppl. materials 3, 4). It differs from *G. humeralis* in skull morphology, with the braincase of *G. lobeke* sp. nov. more robust and broad anteriorly, and with the rostrum also broader and more flattened (Fig. 6A, D); it also differs in the shape of the baculum, which has more robust basal lobes in *G. humeralis* (Fig. 5E, H). As noted above, in *G. humeralis*, the baculum is considered peg-shaped with one lobe, based on a specimen from Uganda (Hill and Harrison 1987). However, in a further specimen from DRC (HZM.1.8023), which compares favourably in cranial, dental, and external morphology to the holotype, and which was collected some 150 km from the type locality, the baculum is shaped like an inverted V but with basal lobes shorter and broader (Fig. 5H). It differs from *G. baka* sp. nov., described above, in its more robust dentition and in the size and shape of the skull, especially the broad, flattened rostrum with its markedly concave dorsal profile (Fig. 6A, E). The baculum of *G. lobeke* sp. nov. is an inverted V-shape as compared to an inverted Y-shape, with expanded basal lobes of *G. baka* sp. nov. (Fig. 5E, I). Finally, the molecular analyses show that *G. lobeke* sp. nov. is phylogenetically close to *G. atra* from which it differs by 4.7% in the Cytb sequences whereas it differs by 13.5% with *G. humeralis* s.s. (Table 4).

##### Distribution and ecology.

The holotype of *G. lobeke* sp. nov. was captured in a triple high net set near Djombi village at an elevation of 570 m a.s.l. This is within a Forest Management Unit (FMU) of the buffer zone of LNP (Fig. 11C). The surrounding area was predominantly small patches of heavily degraded swamp evergreen forest (Fig. 12). The paratype was collected from Yenga fishing site in a harp trap in disturbed forest within the FMU. Additional individuals (based on tissue samples only as the specimens were released) with comparable genetics are included in Hassanin et al. (2018), namely R13-46 and R13-98 from Dzanga-Sangha, southern CAR, and in Torrent et al. (2025a), namely PV917324 (= EBD190113Lo) and PV917326 (= EBD190116) from Ciudad de la Paz, 1°37.01'N, 10°52.83'E in mainland Equatorial Guinea.

### Species accounts for additional *Glauconycteris* species recorded from Cameroon

Based on our field surveys, new data are provided below on a further seven *Glauconycteris* species. There are also brief summaries of two additional species (*G. alboguttata* and *G. poensis*), which have been recorded previously from Cameroon but were not collected as part of our studies. Species are listed in the same order as Table 1.

#### Glauconycteris superba

Taxon classificationAnimaliaChiropteraVespertilionidae

Hayman, 1939

73CFA6F2-7168-5483-8A9A-F88FEA33D18B

##### New material.

Mes 26, ♂ (adult), 27 November 2022, swamp open canopy forest, Mba’a village, buffer zone of DBR, 3°47.71'N, 12°49.52'E, elevation 665 m a.s.l. This is the first record of the species from Cameroon (Fig. 11A). The Cytb sequence showed a 99% similarity with a specimen of *G. superba* from DRC (Hassanin et al. 2018: MF038552) and is available in GenBank (PZ458541) (Suppl. material 1).

##### Status and distribution.

Although only known previously from seven localities in Africa (Monadjem et al. 2024; Torrent et al. 2025a), *G. superba* is listed as LC (Least Concern) by the IUCN Red List based on its wide distribution (Monadjem et al. 2017a). The new locality lies equidistant between two West African records from Ghana and Côte d’Ivoire and the four records from central Africa in DRC and South Sudan (ACR 2024; Monadjem et al. 2024). It complements the new record from ‘Monte Alen’ National Park in mainland Equatorial Guinea (Torrent et al. 2025a).

##### Description of new material.

This is the largest species of *Glauconycteris* (FA: 46.0 mm; Tables 2, 3). The dark brown/black and creamy-white pelage of the Cameroon specimen (Fig. 8A) is comparable in colour and pattern with the holotype of *G. s. sheila* Hayman, 1946, which was collected from Oda in Ghana (5°55.20'N, 0°55.80'W), and with a specimen from South Sudan illustrated in Reeder et al. (2013: fig. 2). The wings are dark without conspicuous reticulation. The ears are angular; each tragus is crescent shaped with a strongly convex, almost semi-circular posterior margin and a slightly concave anterior margin (Fig. 4A). The penis is creamy-white at the base and has a dark, flattened trilobate tip. The baculum is relatively large for *Glauconycteris* (GLB: 1.11 mm) and shaped like an inverted U with a very short distal tip and robust, long, curved basal lobes (Fig. 5A). The skull is robust (Fig. 10A). The first upper incisor (I^2^) is unicuspid; the canines are particularly robust.

##### Ecological notes.

This individual was collected in a mist net in a degraded open canopy swamp forest in DBR, surrounded by unpaved roads, cocoa plantations, and scattered dwellings.

#### Glauconycteris gleni

Taxon classificationAnimaliaChiropteraVespertilionidae

Peterson & Smith, 1973

422946B7-0144-5124-B775-8DAB29D9282C

##### New material.

AD.2024.22.05.222, ♂ (subadult), 22 May 2024, Yenga fishing site, within the Zone of Hunting Interests with Community Management (ZICGC) of LNP, 2°17.59'N, 15°21.37'E, elevation 560 m a.s.l. (Fig. 11A). This study provides the first Cytb sequences for *G. gleni*, which is available in GenBank (PZ458539) (Suppl. material 1). The new Cytb sequence showed an 88% similarity to a specimen of *G. beatrix* (Hassanin et al. 2018: MF038538) and an 87% similarity to a specimen of *G. argentata* (Hassanin et al. 2018: MF038490), both from DRC.

##### Status and distribution.

*Glauconycteris gleni* was previously known from three localities in three countries, Cameroon (type locality: near Lomie, ca 3°10.00'N, 13°37.00'E), Tanzania, and Uganda (Monadjem et al. 2024). Yenga fishing site is now the fourth locality known (Table 1). It is listed as DD (Data Deficient) by the IUCN Red List (Jacobs et al. 2019b). Additionally, two unpublished and unregistered specimens without skulls from Uganda, provisionally referred to this species are held in the Natural History Museum UK (NHMUK). Of these, one from Sango Bay (Field no. 681) may be correctly assigned to *G. gleni* but the latter from Budo (Field no. 682), with a dark brown ventral pelage and very limited reticulation on its wings and interfemoral membrane is not.

##### Description of new material.

This is a larger species of *Glauconycteris* (FA: 41.9 mm in this subadult specimen; Tables 2, 3) characterised by an exceptionally pale, almost pure white ventral pelage. The hairs on the back have fawn-coloured tips and grey bases; there are no pale shoulder spots or lateral flank stripes (Fig. 8B). The wings are pale, with some veins showing, especially adjacent to the body and on the interfemoral membrane, but lacking the marked reticulation of *G. variegata* (Fig. 8C). The face is greyish fawn with a pinkish tinge and with reduced hairs. The ears are sub-rectangular in outline with pronounced margins and a conspicuous semicircular basal lobe adjacent to the mouth (Fig. 8B). Each tragus is almost semi-circular with an evenly rounded posterior margin and almost straight anterior one; it is pale like the ear; the basal lobe is large (Fig. 4B). The penis has a flattened trilobate tip. The baculum (GLB: 0.89 mm) is shaped like an arrowhead, but with the basal lobes deflected downwards, forming a tent-like structure (Fig. 5B). The skull is relatively long (GSL: 13.84 mm in this subadult specimen) but less robust than that of *G. superba* or *G. argentata*. The rostrum is well developed, and the dorsal profile is almost straight (Fig. 10B). The first upper incisor (I^2^) is robust and with a poorly defined secondary cusp. The upper and lower canines are also robust. The lower premolars are not conspicuously reduced.

##### Ecological notes.

This individual was captured in a harp trap set over a stream surrounded by a patch of closed canopy forest far from human habitation within the ZICGC in the buffer zone of LNP. *Glauconycteris lobeke* sp. nov. was collected at the same location.

#### Glauconycteris variegata

Taxon classificationAnimaliaChiropteraVespertilionidae

(Tomes, 1861)

3E977F7D-4BB4-55A8-9145-177D70E64CEA

##### New material.

AD.2024.Z73, ♂ (adult), 24 November 2024, Zega village, within the Zone of Hunting Interests with Community Management (ZICGC) in the buffer zone of LNP, 2°2.22'N, 15°37.73'E, elevation 435 m a.s.l. (Fig. 11A). Morphological evidence was sufficiently robust to identify the species unambiguously.

##### Status and distribution.

*Glauconycteris variegata* is listed as LC (Least Concern) by the IUCN Red List on account of its wide distribution and presumed large population (Monadjem et al. 2017b). It was previously known from two localities in Cameroon (Monadjem et al. 2024). Elsewhere in Africa, there are many records from Angola, Benin, Botswana, CAR, Chad, DRC, Equatorial Guinea, Ethiopia, Gabon, Ghana, Guinea Côte d’Ivoire, Kenya, Malawi, Mozambique, Namibia, Nigeria, Senegal, Sierra Leone, Somalia, South Sudan, Sudan, Tanzania, The Gambia, Uganda, Zambia, and Zimbabwe (Monadjem et al. 2024; Torrent et al. 2025a).

##### Description of new material.

A medium-large species of *Glauconycteris* (FA: 41.2 mm; Tables 2, 3), which is characterised by its yellowish-orange wings which have conspicuous dark brown reticulations (Fig. 8C). The dorsal hairs are whitish at the base, and yellowish or brownish at the tip while the ventral hairs are paler (Fig. 8C). There is no pale spot on the shoulders or lateral flank stripes. The ears are light brown, with rounded tips and a basal fleshy lobe near the mouth. Each tragus is broad and short, with the anterior margin slightly concave and the posterior margin evenly convex (Fig. 4C). The penis has a flattened trilobate tip. Based on Hill and Harrison (1987), the baculum is variable in morphology and size. The one illustrated (Fig. 5C) is from an extralimital specimen from Kenya (HZM.1.1770) and is 1.51 mm in length; shaped like a bottle, the base is curved and hollowed out ventrally. It corresponds to the baculum illustrated in Happold and Happold (2013: fig. 128h) from Mozambique. In the skull, the braincase is robust; the rostrum is short and the dorsal profile weakly concave (Fig. 10C). The first upper incisor (I^2^) is robust and unicuspid.

##### Ecological notes.

This individual was collected in a mist net over a small waterhole in degraded habitat. It was close to an unpaved road, a cocoa plantation, and a settlement within the ZICGC in the buffer zone of LNP.

#### Glauconycteris alboguttata

Taxon classificationAnimaliaChiropteraVespertilionidae

J.A. Allen, 1917

3326CF63-577A-52EC-9F85-660A605355EB

##### New material.

No new material from Cameroon.

##### Status and distribution.

Despite being little known, with just 15 localities listed from six African countries (Cameroon, Equatorial Guinea, CAR, DRC, Republic of Congo, and Uganda) (Monadjem et al. 2024; Torrent et al. 2025a), *G. alboguttata* is listed as LC (Least Concern) by the IUCN Red List on account of its wide distribution (Monadjem et al. 2017c). In Cameroon, it is known from six localities (Monadjem et al. 2024) (Fig. 11B).

##### Description.

A medium-sized species (FA: 38.0–42.0 mm; Table 1). The pelage is sepia-brown with a clearly defined pale spot on each shoulder and a pale stripe on each flank; the wings are brown and without conspicuous reticulation (Hassanin et al. 2018: fig. 6). Each ear is rounded, and the tragus is broad, with the outer margin evenly convex to the tip (Fig. 4D). No data are available on the baculum. The skull, based on the holotype from DRC, has a broad flattened rostrum; the dorsal profile is slightly concave (Fig. 10D). The first upper incisor is bicuspid.

##### Ecological notes.

There are no new data on the ecology of this species in Cameroon.

#### Glauconycteris argentata

Taxon classificationAnimaliaChiropteraVespertilionidae

(Dobson, 1875)

4AB94CCB-94B3-5903-8E28-3C3B30F0B444

##### New material.

MAN.177, ♂ (adult), 21 December 2019, Mbouroukou école publique, Mount Manengouba Herpeto-ornithological Sanctuary, 5°2.15'N, 9°51.96'E, elevation 1899 m a.s.l.; previously included, but without details, in Bakwo-Fils et al. (2021). The Cytb sequence showed a 100% similarity with a specimen of *G. argentata* from Cameroon (Grunwald et al. 2025: PP942856), and is available in GenBank (PZ458542) (Suppl. material 1).

##### Status and distribution.

This is a relatively widespread and abundant species in Central and East Africa and is considered LC (Least Concern) by the IUCN Red List (Monadjem et al. 2017e). The new locality is additional to the 12 from Cameroon, including the type locality (Mount Cameroon), listed in Monadjem et al. (2024) (Fig. 11B). Elsewhere in Africa, it is recorded from Angola, Burundi, DRC, Equatorial Guinea, Kenya, Malawi, Tanzania, Republic of Congo, Rwanda, and Uganda (Monadjem et al. 2024; Torrent et al. 2025a).

##### Description of new material.

A medium-sized species of *Glauconycteris* (FA: 42.2 mm; Tables 1, 2, 3), with a characteristically pale fawn dorsal pelage, and a pale lateral stripe on each flank (Fig. 8D). The hairs on the back are bicoloured, beige with brown at the base and golden fawn to greyish brown at the tip; there is no white shoulder spot. The wings are pale with some reticulation. Unlike the rectangular and bicoloured ears of *G. egeria* (Grunwald et al. 2025: fig. 4Z), the ears are rounded and pale (Fig. 8D). Each tragus is broad with an anterior margin slightly concave and a posterior margin evenly convex (Fig. 4E). The penis has a flattened trilobate tip. The baculum (GLB: 0.87 mm) is shaped like an arrowhead; the basal lobes are clearly defined and deflected downwards (Table 2, Fig. 5D). The skull has a short, reduced rostrum and an almost straight dorsal profile (Fig. 9A). The first upper incisor (I^2^) has a weak secondary cusp.

##### Ecological notes.

This specimen was one of 13 individuals captured on Mount Manengouba at 1900 m a.s.l. All were taken in mist nets placed over slow-flowing streams in a pocket of riparian forest in an area that had been degraded by overgrazing (Bakwo-Fils et al. 2021). Elsewhere in Cameroon, in 2019, five individuals (four males and one female) were collected at one location on Mt Mbam Minkom in mist nets placed over streams (Grunwald et al. 2025). In DBR, two individuals were collected in mist-nets set at ground level in primary forest (Atagana et al. 2021).

#### Glauconycteris egeria

Taxon classificationAnimaliaChiropteraVespertilionidae

Thomas, 1913

A210E2C1-B859-500E-98DC-6475234B3118

##### New material.

Mes 38, ♀ (adult), 29 November 2022, Blandjock village, in the buffer zone of DBR, 3°47.14'N, 12°50.21'E, elevation 680 m a.s.l. The Cytb sequence showed a 99% similarity with a specimen of *G. egeria* from CAR (Hassanin et al. 2018: MF038546) (Suppl. material 1).

##### Status and distribution.

*Glauconycteris egeria* is listed as DD (Data Deficient) by the IUCN Red List based on insufficient information on its extent of occurrence, natural history, threats, and conservation status (Jacobs et al. 2019a). In Cameroon, it was previously known from five localities including the type locality of Bibundi (Monadjem et al. 2024) (Fig. 11C). A specimen from the southern part of Mpem and Djim National Park (Atagana et al. 2018) and an individual from Centre Region of Cameroon, but without details (Waghiiwimbom et al. 2019) were omitted by Monadjem et al. (2024). Elsewhere in Africa, it is known from a further seven localities in the following countries: Equatorial Guinea, CAR, Ghana, Nigeria, and Uganda (Monadjem et al. 2024; Torrent et al. 2025a).

##### Description of new material.

A medium-sized species of *Glauconycteris* (FA: 40.0 mm; Tables 2, 3) with dark brown dorsal and ventral pelage and a pale lateral stripe on each flank. The ears are characteristically angular and bicoloured with pale borders and a dark inner conch (Hassanin et al. 2018: fig. 6; Grunwald et al. 2025: fig. 4Z). Each tragus is crescent shaped with a convex posterior margin and a moderately concave anterior margin (Fig. 4F). No data are available on the penis or baculum. The skull is robust (GSL: 13.72 mm), and the dorsal profile is concave (Fig. 9B). The anterior upper incisor (I^2^) is weakly bicuspid with the secondary cusp defined but small, there is a third cusplet visible on the cingulum; the upper canines are robust.

##### Ecological notes.

This individual was collected in a mist net in degraded open canopy swamp forest in the buffer zone of DBR. Elsewhere, one individual was captured in a mist net in undisturbed montane forest, between 1,601 to 1,800 m asl., on Mount Cameroon (Mongombe et al. 2019; Bakwo-Fils et al. 2021). A male specimen was collected in a mist net set in a forest beside a river in the southern part of Mpem and Djim National Park (Atagana et al. 2018). An individual from Centre Region was captured in forest, but without further details (Waghiiwimbom et al. 2019).

#### Glauconycteris curryae

Taxon classificationAnimaliaChiropteraVespertilionidae

Eger & Schlitter, 2001

B9300CA6-2490-56B6-9D12-226397B824D5

##### New material.

AD.2023.11.25.181, ♂ (adult), 25 November 2023, intact forest, Pont cassé platform, in the core area of LNP, 2°17.19'N, 15°40.51'E, elevation 440 m a.s.l. AD.2023.11.28.196, 1♀ (adult), 28 November 2023, intact forest, near Bai petite savane platform, in the core area of LNP, 2°17.72'N, 15°42.76'E, elevation 420 m a.s.l. The two Cytb sequences showed a 98–99% similarity with a specimen of *G. curryae* from DRC (Hassanin et al. 2018: MF038505) and are available in GenBank (PZ458537; PZ458538) (Suppl. material 1).

##### Status and distribution.

*Glauconycteris curryae* is listed as DD (Data Deficient) by the IUCN Red List on account of the limited information known about its occurrence, status, threats, and ecological requirements (Schlitter 2019). In Cameroon, it was previously known from five localities including Bipindi, the type locality (Monadjem et al. 2024) (Fig. 11C). Elsewhere in Africa, it is known from 11 localities in four countries: CAR, DRC, Equatorial Guinea, and Gabon (Monadjem et al. 2024; Torrent et al. 2025a).

##### Description of new material.

A medium-small species of *Glauconycteris* with a short forearm (FA: 35.1, 37.9 mm; Table 2) but a robust skull (GSL: 12.4 mm; Table 3). The dorsal pelage is characteristically orange-brown; the ventral pelage is rufous-brown. The dorsal hairs are conspicuously tricoloured, the basal half is blackish, followed by a narrow band of whitish buff, and brownish at the tip. Neither specimen has a pale spot on the shoulder or lateral flank stripes (Fig. 8E). The wings are dark without reticulation. There is a black contour surrounding each eye. The ears are slightly elongated with a rounded tip. Each tragus has a straight anterior margin and an almost straight posterior margin with a rounded tip (Fig. 4H). The penis has a flattened trilobate tip. The baculum is particularly small (GLB: 0.55 mm) (Table 2) and shaped like an inverted Y with distal shaft and basal lobes equally developed (Fig. 5F). In the adult male, the skull is partly damaged, with a CCL of 11.11 mm, which is slightly shorter than the female (CCL: 11.49 mm). In both skulls the braincase is bulbous, and the dorsal profile is distinctly concave (Fig. 9C). The anterior upper incisor (I^2^) is bicuspid with the secondary cusp well defined.

##### Ecological notes.

Both specimens were captured in mist nets in the central core area of LNP. The male was collected, together with *G. beatrix* (see below), over a stream within closed canopy swamp forest; the female was collected over a water course also in closed canopy forest. Elsewhere in Cameroon, the holotype, a pregnant female, was netted at 300 m a.s.l., 10 km west of Bipindi, over a pool in a vast stand of bamboo by a river; an additional female specimen was netted over a river pool near the south shore of Lake Barombi and another female netted at 460 m asl. over a forest stream, 67 km west of Ayos (Eger and Schlitter 2001).

#### Glauconycteris poensis

Taxon classificationAnimaliaChiropteraVespertilionidae

(Gray, 1842)

06CE49EF-1DA9-5EFA-AB36-ADC0F1399852

##### New material.

There is no new material from Cameroon.

##### Status and distribution.

*Glauconycteris poensis* is listed as LC (Least Concern) by IUCN based on its wide distribution and presumed large population (Monadjem et al. 2017d). However, in Cameroon it is relatively rare, recorded from just two localities (Monadjem et al. 2024) (Fig. 11D). Elsewhere in Africa, it is known from Benin, CAR, DRC, Equatorial Guinea, Ghana, Guinea, Côte d’Ivoire, Liberia, Nigeria, Senegal, Sierra Leone, Southern Sudan, Tanzania, and Togo (Monadjem et al. 2024; Torrent et al. 2025a).

##### Description.

A medium-small species of *Glauconycteris* (FA: 32–41 mm), with females larger than males (Happold and Happold 2013) (Table 1). The dorsal pelage is medium to dark greyish brown and there is usually a stripe on each flank and a pale spot on each shoulder (Hassanin et al. 2018: fig. 6), although sometimes one or both are absent. The wings are dark without conspicuous reticulation. The ears are rounded; each tragus has a concave anterior margin and a convex posterior margin (Happold and Happold 2013: fig. 124e). The baculum is variable but essentially shaped like an inverted ‘V’ (Happold and: fig. 128e, f). The skull has bulbous braincase, and the dorsal profile is distinctly concave (Fig. 9D). The anterior upper incisor (I^2^) is weakly bicuspid.

##### Ecological notes.

There are no new data on the ecology of this species in Cameroon.

#### Glauconycteris beatrix

Taxon classificationAnimaliaChiropteraVespertilionidae

Thomas, 1901

235F963A-F4E7-5D45-A80E-567E9A05FC42

##### New material.

AD.2023.11.25.182, ♂ (adult), 25 November 2023, intact forest, Pont cassé platform, in the core area of LNP, 2°17.23'N, 15°40.50'E, elevation 436 m a.s.l. The Cytb sequence showed a 99% similarity with a specimen of *G. beatrix* from Cameroon (Hassanin et al. 2018: MF038540) and is available in GenBank (PZ425899) (Suppl. material 1).

##### Status and distribution.

*Glauconycteris beatrix* is listed as LC (Least Concern) by IUCN in view of its wide distribution (Monadjem et al. 2017f). Monadjem et al. (2024) included it from six localities in Cameroon (Fig. 11D), and 29 localities in nine countries elsewhere in Africa: Angola, CAR, DRC, Equatorial Guinea, Ghana, Côte d’Ivoire, Nigeria, Republic of Congo, and Sierra Leone. Torrent et al. (2025a) contributed a further four localities from Equatorial Guinea. In the light of current taxonomic research (Hassanin et al. 2018 and this study), this distribution will have to be reassessed.

##### Description of new material.

This is a small species of *Glauconycteris* (FA: 36.3 mm; Tables 2, 3). The dorsal pelage is a deep brown with a pale spot on each shoulder and no lateral flank stripe (Fig. 3B). The wings are mid to dark brown without conspicuous reticulation. The ears are rounded (Fig. 3B). Each tragus has an almost straight anterior margin and an evenly rounded convex posterior margin (Fig. 4I). The penis has a flattened trilobate tip. The baculum is small (GLB: 0.57 mm), ‘V’-shaped with the two long, slender basal arms (Fig. 5G, Table 2). The skull (GSL: 11.56 mm) has a well-developed rostrum and a slightly concave profile (Fig. 6B, C). The anterior upper incisor (I^2^) is bicuspid with the secondary cusp well defined; the canines are moderately developed (Fig. 7B).

##### Ecological notes.

Together with *G. curryae*, the individual was captured in a mist net over a stream within a closed canopy swamp forest in the central core area of LNP.

## Discussion

Building on the comprehensive revision of *Glauconycteris* by Hassanin et al. (2018), our results refine the systematic framework of the genus and reveal previously unrecognised diversity. Hassanin et al. (2018) combined morphological data with molecular phylogenetic analyses to produce a reconstruction that confirmed most previously described taxa. That analysis identified a basal split separating *G. superba* and *G. variegata* from the remaining species, which form a large monophyletic clade comprising three species groups: *poensis*, *beatrix*, and *humeralis*. That study also described a new species, *G. atra*, but left several systematic questions unresolved and highlighted a number of still-unnamed lineages within these major groupings.

In the present study, we confirm the overall phylogenetic framework proposed by Hassanin et al. (2018) and refine it further. Our results demonstrate that *G. beatrix* sensu Hassanin et al. (2018) represents a species complex, within which *G. baka* sp. nov. is nested. Meanwhile, *G. lobeke* sp. nov. (= *G. cf. humeralis* sensu Hassanin et al. 2018) is nested within the *humeralis* species group. These findings contrast sharply with earlier studies that tended to synonymise taxa rather than recognise their diversity. For example, *G. humeralis* (together with *G. alboguttata*) was once regarded as conspecific with *G. poensis* (Hayman and Jones 1950; Koopman 1965), while others considered it a synonym of *G. beatrix* (Koopman 1971; Heller et al. 1994; Monadjem et al. 2020a). Conversely, Rosevear (1965) and Happold (1987) proposed that *G. beatrix* was conspecific with *G. poensis*.

These conflicting arrangements illustrate the considerable challenges taxonomists have faced in resolving relationships within *Glauconycteris* based solely on morphological characters, further complicated by size differences between males (smaller) and females (larger) in some taxa (Happold and Happold 2013). Such difficulties likely reflect a degree of morphological conservatism and convergence in fur coloration and spotting patterns, traits that have traditionally been used as diagnostic. In this regard, molecular approaches have proven invaluable for clarifying evolutionary relationships and supporting the recognition of new taxa, such as *G. baka* sp. nov. and *G. lobeke* sp. nov. Considered within the evolutionary framework of Hassanin et al. (2018), it is likely that undescribed taxa remain to be discovered, not only within the *beatrix* and *humeralis* species groups, but also within the *poensis* group (e.g. *G. egeria* and/or *G. argentata*) and potentially within the *G. variegata* clade.

The taxonomic findings of our study have important implications for the conservation of *Glauconycteris*, particularly in relation to species distributions, conservation status, and occurrence within protected areas. Our revised species list confirms that all 11 *Glauconycteris* species known from Cameroon have been recorded within at least one protected area (PA) or its buffer zone (Table 5). Notably, *G. superba* is reported here for the first time from Cameroon, based on a specimen from the buffer zone of DBR. This represents the eighth known locality for the species in Africa. This geographically intermediate record, linking two sites in Côte d’Ivoire and Ghana with three in DRC and South Sudan (ACR 2024), complements the recent mainland Equatorial Guinea record reported by Torrent et al. (2025a) and further supports the view that this spectacular species is more widespread than previously recognised, consistent with its current IUCN Red List classification as Least Concern (LC) (Monadjem et al. 2017a).

**Table 5.** Ecological and conservation data for all species of *Glauconycteris*. Species order follows Table 1.

Species (IUCN status) Records from protected areas in Cameroon (bz = buffer zone)	Habitat of material captured for this study in Cameroon	Ecological notes from previous studies throughout Africa
*G. superba* (LC) Dja Biosphere Reserve (bz)	Degraded open canopy swamp forest	Secondary forest (Gembu Tungaluna et al. 2013); grassland plateau above secondary thicket forest (Reeder et al. 2013); semi-deciduous forest (ca. 360 m asl); evergreen lowland forest (ca. 190 m asl) NE Congolian lowland rainforest (ca. 700 m) (Happold and Happold 2013); young secondary forest in stagnant water; primary forest (Ing et al. 2016)
*G. gleni* (DD) Lobéké NP (bz); Dja Biosphere Reserve	Patch of closed canopy forest	Tropical forest (Peterson and Smith 1973); rainforest (Happold and Happold 2013)
*G. variegata* (LC) Lobéké NP (bz)	Degraded forest with settlements and cocoa plantations	Savannah woodland or open-country-bushveldt habitat; riverine forest; dense riparian forest (Rambaldini 2010); occasionally degraded rainforest (Rosevear 1965)
*G. machadoi* (DD)	na	Wetter miombo woodland dominated by *Brachystegia* and *Julbernardia* (Happold and Happold 2013)
*G. alboguttata* (LC) Mount Cameroon NP; Dja Biosphere Reserve	na	Rainforest (Happold and Happold 2013); Okoumé (*Aucoumea klaineana*) evergreen forest bordering forest-savannah mosaic (Bates et al. 2013)
*G. argentata* (LC) Dja Biosphere Reserve; Mount Cameroon NP; Mount Manengouba Sanctuary; Rumpi Hills Protected Area	Riparian forest in degraded habitat in Mount Manengouba	Coconut and oil palms; border of forest and Guinean woodland zones; drier country (Rosevear 1965); forests at high and low altitude; in East Africa found in areas in excess of 900 mm annual rainfall (Kingdon 1974); bamboo forest; miombo woodland; coastal forests in Tanzania (Happold and Happold 2013); disturbed habitat (Bakwo-Fils et al. 2021); primary forest (Atagana et al. 2021); undisturbed and disturbed forest (Grunwald et al. 2025)
*G. kenyacola* (DD)	na	Possibly restricted to lower coastal plains of Kenya and adjacent regions (Peterson 1982)
*G. egeria* (DD) Mount Cameroon NP; Dja Biosphere Reserve	Degraded open canopy swamp forest	Dense secondary tropical lowland forest (Lunde et al. 2001); ‘forest’ (Waghiiwimbom et al. 2019); undisturbed Afromontane forest between 1,601 to 1,800 m asl (Mongombe et al. 2019; Bakwo-Fils et al. 2021); riparian forest (Atagana et al. 2021); dense tropical and secondary forest; forest-savanna mosaic (Grunwald et al. 2025)
*G. atra* (na)	na	Riparian ‘zones’ on left bank of Congo River (Hassanin et al. 2018)
*G. lobeke* sp.nov. (na) Lobéké NP (bz)	Degraded swamp evergreen forest	na
*G. curryae* (DD) Lobéké NP	Intact closed canopy swamp forest	Rainforest; bamboo stands; forests alongside lake (Happold and Happold 2013); 300-460 m asl (Eger and Schlitter 2001)
*G. poensis* (LC) Douala-Edea WR; Mount Cameroon NP (adjacent to)	na	Rainforest; primary forest surrounded by farmland; cocoa plantations up to 1300 m asl; swamp forest (Happold and Happold 2013); forested and degraded secondary habitats, 450–800 m asl (Monadjem et al. 2016); lowland tropical moist forest (ACR 2024)
*G. beatrix* (LC) Lobéké NP; Mount Cameroon NP (adjacent to)	Intact closed canopy swamp forest	Lowland rainforest; rainforests outside rainforest zone; secondary forest, cocoa plantations; swamp forest (Happold and Happold 2013); Oukoumé (*Aucoumea klaineana*) evergreen forest bordering forest savannah mosaic (Bates et al. 2013)
*G. humeralis* (DD) na	na	Rainforest, including areas of grassy glades in extensive stands of evergreen forest (Happold and Happold 2013)
*G. baka* sp. nov. (na) Lobéké NP (bz)	Open canopy evergreen Congolian lowland forest	na

In this context, other species, such as *G. beatrix* and *G. humeralis*, which are currently considered relatively widespread (ACR 2024), will require reassessment for the IUCN Red List in the light of present findings and ongoing taxonomic revisions. It will also be essential to refine the known distributions of newly described and poorly known taxa, especially since several species, including *G. kenyacola* and *G. machadoi*, are currently restricted to their type localities, while others (*G. alboguttata*, *G. atra*, *G. baka* sp. nov., *G. curryae*, *G. egeria*, *G. gleni*, *G. humeralis*, *G. lobeke* sp. nov., and *G. superba*) are each represented by relatively few known localities (Table 1). Addressing these gaps will involve the re-examination of museum collections as well as targeted field surveys.

We have provided new distributional data for three of the six Data Deficient *Glauconycteris* species (*G. curryae*, *G. egeria*, and *G. gleni*). The record of *G. curryae* from LNP links verified populations in western and southern Cameroon with those from mainland Equatorial Guinea and further east in DRC (ACR 2024; Torrent et al. 2025a). For *G. gleni*, the new record represents only the fourth published locality for Africa, but supports the view that this species may have a broad distribution across the Congolian Basin. A similar pattern of wide but poorly documented occurrence applies to *G. egeria*.

In terms of conservation, *G. atra* and *G. kenyacola* are not currently represented within any protected area (PA) across sub-Saharan Africa, and several species, including *G. superba*, *G. gleni*, *G. machadoi*, *G. lobeke* sp. nov. (= *G. cf. humeralis*), and *G. curryae*, are known from only a very limited number (Montauban et al. 2025). However, species distribution models suggest that the actual number of protected areas supporting *Glauconycteris* species is likely to be substantially higher. For example, *G. curryae* is predicted to occur in 252 PAs (Montauban et al. 2025).

In addition, our ecological observations provide useful insights into the habitat requirements of *Glauconycteris*. Only two species (*G. beatrix* and *G. curryae*) were captured in relatively undisturbed forest, both within the core zone of LNP. The remaining six were recorded in degraded habitats within the buffer zones of LNP and DBR, although *G. gleni*, together with the paratype of *G. lobeke* sp. nov., was captured in a remnant patch of closed-canopy forest distant from human settlements. When considered more broadly, available data suggest that most *Glauconycteris* species can tolerate at least some degree of habitat disturbance (Table 5). Together, these distributional and ecological patterns provide important context for interpreting the conservation status of *Glauconycteris* species across the region.

In conclusion, our study further confirms the exceptional bat diversity of Cameroon with more than 120 bat species (ACR 2024; Grunwald et al. 2025), and demonstrates that even well-recognised genera such as *Glauconycteris* continue to harbour substantial, previously unrecognised diversity. The discovery of two new species from a limited sampling effort, together with evidence of additional cryptic lineages, underscores the extent to which the diversity of African forest bats remains underestimated.

These findings highlight the importance of the Guineo-Congolian forest block as a major centre of diversity and ongoing evolutionary diversification for *Glauconycteris*, where further undescribed taxa are likely to occur. At the same time, they emphasise the need for integrative approaches that combine molecular, morphological, and ecological data to resolve species boundaries and refine conservation assessments.

In this context, continued baseline surveys in the Northwestern Congolian Lowland Forest, particularly in poorly explored areas, such as Lobéké National Park, are essential, not only to document biodiversity, but to ensure that conservation strategies are based on an accurate understanding of species diversity, distribution, and ecological resilience in one of Africa’s most important forest systems.

## Supplementary Material

XML Treatment for Glauconycteris baka

XML Treatment for Glauconycteris lobeke

XML Treatment for Glauconycteris superba

XML Treatment for Glauconycteris gleni

XML Treatment for Glauconycteris variegata

XML Treatment for Glauconycteris alboguttata

XML Treatment for Glauconycteris argentata

XML Treatment for Glauconycteris egeria

XML Treatment for Glauconycteris curryae

XML Treatment for Glauconycteris poensis

XML Treatment for Glauconycteris beatrix

## Additional information

### Artificial Intelligence (AI) use

The authors accept full responsibility for the content of the manuscript, including the disclosure of any use of AI.

No AI tools were used in the preparation of this manuscript.

### Author ORCIDs

A. Gomeh-Djame https://orcid.org/0009-0002-0914-2905

P. J.J. Bates https://orcid.org/0000-0003-3630-739X

J. Juste https://orcid.org/0000-0003-1383-8462

M. Suarez-Rubio https://orcid.org/0000-0002-0596-2626

L. Torrent https://orcid.org/0000-0001-5036-6359

J.L. García-Mudarra https://orcid.org/0000-0003-0561-9323

E. A. Ayangma https://orcid.org/0009-0002-5001-7583

F. Yettore https://orcid.org/0009-0002-1295-2749

C.F.B. Bilong https://orcid.org/0000-0003-0138-5718

E.-M. Bakwo-Fils https://orcid.org/0000-0001-7878-8158

### Data availability

All sequences generated in this study have been deposited in the GenBank.

## Supplementary materials

Supplementary material 1 Supplementary information 1 Data type docx Explanation note Concatenated mitochondrial DNA (mtDNA) sequences including Cytb, COI and 12S, and nuclear DNA (nuDNA) sequences including HDAC2, RAG2, RIOK3, ZFYVE27, ABHD11, ACOX2, COPS7A-4, ROGDI, ACTP1, STAT5 of bat species from the genus *Glauconycteris* generated and used in this study. Suffixes H1 and H2 indicate distinct haplotypes. For each sequence, we provide the species name, the specimen identification number (museum number or field number) as it appears in our phylogenetic trees (Fig. 2, Suppl. materials 3, 4), the corresponding GenBank accession number, the locality of collection, the type of molecular marker (mtDNA or nuDNA), and the authorship of the sequence. All sequences from the current study have been deposited in GenBank. https://binary.pensoft.net/file/1678582This dataset is made available under the Open Database License (http://opendatacommons.org/licenses/odbl/1.0/). The Open Database License (ODbL) is a license agreement intended to allow users to freely share, modify, and use this Dataset while maintaining this same freedom for others, provided that the original source and author(s) are credited. Aicha Gomeh-Djame, Paul J. J. Bates, Javier Juste, Marcela Suarez-Rubio, Laura Torrent, Juan Luis García-Mudarra, Eugenie Agodigo Ayangma, Félécité Yettore, Charles Félix Bilong Bilong, Eric-Moise Bakwo-Fils

Supplementary material 2 Supplementary information 2 Data type xlsx Explanation note Definitions of external and craniodental measurements (in mm). Definitions follow Bates and Harrison (1997). https://binary.pensoft.net/file/1678583This dataset is made available under the Open Database License (http://opendatacommons.org/licenses/odbl/1.0/). The Open Database License (ODbL) is a license agreement intended to allow users to freely share, modify, and use this Dataset while maintaining this same freedom for others, provided that the original source and author(s) are credited. Aicha Gomeh-Djame, Paul J. J. Bates, Javier Juste, Marcela Suarez-Rubio, Laura Torrent, Juan Luis García-Mudarra, Eugenie Agodigo Ayangma, Félécité Yettore, Charles Félix Bilong Bilong, Eric-Moise Bakwo-Fils

Supplementary material 3 Supplementary image 1 Data type png Explanation note Bayesian phylogenetic reconstruction based on the concatenation of mitochondrial genes (Cytb, 12S, COI). Values above branches represent Bayesian posterior probabilities and values below branches are bootstrap values of Maximum Likelihood (support values < 75 are not shown). The outgroup taxon was *Glauconycteris superba*. https://binary.pensoft.net/file/1678584This dataset is made available under the Open Database License (http://opendatacommons.org/licenses/odbl/1.0/). The Open Database License (ODbL) is a license agreement intended to allow users to freely share, modify, and use this Dataset while maintaining this same freedom for others, provided that the original source and author(s) are credited. Aicha Gomeh-Djame, Paul J. J. Bates, Javier Juste, Marcela Suarez-Rubio, Laura Torrent, Juan Luis García-Mudarra, Eugenie Agodigo Ayangma, Félécité Yettore, Charles Félix Bilong Bilong, Eric-Moise Bakwo-Fils

Supplementary material 4 Supplementary image 2 Data type png Explanation note Bayesian phylogenetic reconstruction based on the concatenation of six nDNA markers ABHD11, ACOX2, COPS7A-4, ROGDI, ACTP1, STAT5) that were amplified for a subset of the samples See text for the trees’ details. Values above branches represent Bayesian posterior probabilities and values below branches are bootstrap values after 1000 iterations in the reconstruction under a Maximum Likelihood (support values < 75 are not shown). https://binary.pensoft.net/file/1678585This dataset is made available under the Open Database License (http://opendatacommons.org/licenses/odbl/1.0/). The Open Database License (ODbL) is a license agreement intended to allow users to freely share, modify, and use this Dataset while maintaining this same freedom for others, provided that the original source and author(s) are credited. Aicha Gomeh-Djame, Paul J. J. Bates, Javier Juste, Marcela Suarez-Rubio, Laura Torrent, Juan Luis García-Mudarra, Eugenie Agodigo Ayangma, Félécité Yettore, Charles Félix Bilong Bilong, Eric-Moise Bakwo-Fils
