# Supplementary material for: An integrated taxonomic and conservation assessment of Glauconycteris (Chiroptera, Vespertilionidae) in Cameroon, with the description of two new species from the Northwestern Congolian Lowland Forest
Source: Zookeys. 2026 Jun 15;1282:161–204. doi: 10.3897/zookeys.1282.183038 (PMC13288025; doi:10.3897/zookeys.1282.183038)
Supplement: Supplementary material 1 — Supplementary information 1 [file zookeys-1282-161_article-183038__-s001.docx]

Table S1. Concatenated mitochondrial DNA (mtDNA) sequences including Cytb, COI and 12S, and nuclear DNA (nuDNA) sequences including HDAC2, RAG2, RIOK3, ZFYVE27, ABHD11, ACOX2, COPS7A-4, ROGDI, ACTP1, STAT5 of bat species from the genus *Glauconycteris* generated and used in this study. Suffixes H1 and H2 indicate distinct haplotypes. For each sequence, we provide the species name, the specimen identification number (museum number or field number) as it appears in our phylogenetic trees (Figure 2, Figure S1, Figure S2), the corresponding GenBank accession number, the locality of collection, the type of molecular marker (mtDNA or nuDNA), and the authorship of the sequence.

| **Species** | **Identification number** | **GenBank accession numbers** | **mtDNA markers (**COI, CYTB, 12S**)** | **nuDNA markers** (HDAC2, RIOK3, ZFYVE27, RAG2) | **nuDNA markers** (ACOX2, ABHD11, COPS, RODGI, ACTP1, STAT5) | **Locality** | **Reference** |
| --- | --- | --- | --- | --- | --- | --- | --- |
| *Glauconycteris superba* | MNHN2016-2803 *G. superba* | MF038653 (COI); MF038552 (Cytb); MF038750 (12S). | X | X | - | Mbiye, DRC | Hassanin et al. 2018 |
| *Glauconycteris superba* | Mes26 *G. superba* | PZ458541 (Cytb); PZ458462 (STAT); PZ458518 (ACTP); PZ458564 (RODGI);  PZ468907 (ACOX2);  PZ468947 (ABHD11) | X | - | X | Dja Biodiversity Reserve, Cameroon | This study |
| *Glauconycteris superba* | EBD200204Su *G. superba* | PZ458448 (STAT); PZ458486 (COPS); PZ458549 (RODGI);  PZ468906 (ACOX2);  PZ468934 (ABHD11) | - | - | X | Mainland, Equatorial Guinea | This study |
| *Glauconycteris egeria* | R13-44 *G. egeria* | MF038647 (COI); MF038546 (Cytb); MF038747 (12S) | X | X | - | Dzanga-Sangha, CAR | Hassanin et al. 2018 |
| *Glauconycteris egeria* | R13-48 *G. egeria* | MF038648 (COI); MF038547 (Cytb); MF038748 (12S) | X | X | - | Dzanga-Sangha, CAR | Hassanin et al. 2018 |
| *Glauconycteris egeria* | Mes38 *G. egeria* | PZ458540 (Cytb); PZ458461 (STAT); PZ458500 (COPS); PZ458517 (ACTP); PZ458563 (RODGI);  PZ468910 (ACOX2);  PZ468946 (ABHD11) | X | - | X | Dja Biodiversity Reserve, Cameroon | This study |
| *Glauconycteris egeria* | EBD200129 *G. egeria* | PZ458483 (COPS); PZ458502 (ACTP_H1); PZ458503 (ACTP_H2); PZ458546 (RODGI);  PZ468911 (ACOX2);  PZ468932 (ABHD11) | - | - | X | Mainland, Equatorial Guinea | This study |
| *Glauconycteris alboguttata* | MNHN2016-2775 *G. alboguttata* | MF038613 (COI); MF038512 (Cytb); MF038713 (12S) | X | X | - | Melume, DRC | Hassanin et al. 2018 |
| *Glauconycteris alboguttata* | R13-31 *G. alboguttata* | MF038617 (COI); MF038516 (Cytb); MF038717 (12S) | X | X | - | Dzanga-Sangha, CAR | Hassanin et al. 2018 |
| *Glauconycteris alboguttata* | R13-55 *G. alboguttata* | MF038621 (COI); MF038520 (Cytb); MF038721 (12S) | X | X | - | Dzanga-Sangha, CAR | Hassanin et al. 2018 |
| *Glauconycteris alboguttata* | EBD200204Al *G. alboguttata* | PZ458447 (STAT); PZ458484 (COPS_H1); PZ458485 (COPS_H2); PZ458504 (ACTP); PZ458547 (RODGI_H1); PZ458548 (RODGI_H2);  PZ468912 (ACOX2_H1);  PZ468913 (ACOX2_H2); PZ468933 (ABHD11) | - | - | X | Mainland, Equatorial Guinea | This study |
| *Glauconycteris atra* | MNHN2016-2790 *G. atra* | MF038600 (COI); MF038499 (Cytb); MF038700 (12S) | X | X | - | Yatolema, DRC | Hassanin et al. 2018 |
| *Glauconycteris atra* | MNHN2016-2791 *G. atra* | MF038601 (COI); MF038500 (Cytb); MF038701 (12S) | X | X | - | Yaengo, DRC | Hassanin et al. 2018 |
| *Glauconycteris atra* | MNHN2016-2792 *G. atra* | MF038602 (COI); MF038501 (Cytb); MF038702 (12S) | X | X | - | Yaengo, DRC | Hassanin et al. 2018 |
| *Glauconycteris atra* | MNHN2016-2793 *G. atra* | MF038603 (COI); MF038502 (Cytb); MF038703 (12S) | X | X | - | Yatolema, DRC | Hassanin et al. 2018 |
| *Glauconycteris argentata* | MNHN2016-2779 *G. argentata* | MF038586 (COI); MF038485 (Cytb); MF038686 (12S) | X | X | - | Melume, DRC | Hassanin et al. 2018 |
| *Glauconycteris argentata* | MNHN2016-2780 *G. argentata* | MF038587 (COI); MF038486 (Cytb); MF038687 (12S) | X | X | - | Mbiye, DRC | Hassanin et al. 2018 |
| *Glauconycteris argentata* | EBD230113 *G. argentata* | PZ458450 (STAT); PZ458488 (COPS); PZ458506 (ACTP); PZ458551 (RODGI);  PZ468909 (ACOX2);  PZ468936 (ABHD11) | - | - | X | Mainland, Equatorial Guinea | This study |
| *Glauconycteris argentata* | MAN177 *G. argentata* | PZ458542 (Cytb) | X | - | - | Mount Manengouba Herpeto-ornithological Sanctuary, Cameroon | This study |
| *Glauconycteris curryae* | MNHN2016-2795 *G. curryae* | MF038605 (COI); MF038504 (Cytb); MF038705 (12S) | X | X | - | Bongandjola, DRC | Hassanin et al. 2018 |
| *Glauconycteris curryae* | MNHN2016-2796 *G. curryae* | MF038606 (COI); MF038505 (Cytb); MF038706 (12S) | X | X | - | Yaengo, DRC | Hassanin et al. 2018 |
| *Glauconycteris curryae* | R13-115 *G. curryae* | MF038610 (COI); MF038509 (Cytb); MF038710 (12S) | X | X | - | Dzanga-Sangha, CAR | Hassanin et al. 2018 |
| *Glauconycteris curryae* | 20231125181 *G. curryae* | PZ458537 (Cytb); PZ458458 (STAT); PZ458496 (COPS); PZ458514 (ACTP); PZ458559 (RODGI);  PZ468915 (ACOX2);  PZ468943 (ABHD11) | X |  | X | Lobéké National Park, Cameroon | This study |
| *Glauconycteris curryae* | 20231128196 *G. curryae* | PZ458538 (Cytb); PZ458459 (STAT_H1); PZ458460 (STAT_H2); PZ458497 (COPS); PZ458515 (ACTP); PZ458560 (RODGI);  PZ468916 (ACOX2);  PZ468944 (ABHD11) | X |  | X | Lobéké National Park, Cameroon | This study |
| *Glauconycteris curryae* | EBD190113Cu *G. curryae* | PZ458445 (STAT_H1); PZ458446 (STAT_H2); PZ458482 (COPS); PZ458501 (ACTP); PZ458545 (RODGI);  PZ468914 (ACOX2);  PZ468931 (ABHD11) | X |  | X | Mainland, Equatorial Guinea | This study |
| *Glauconycteris beatrix* | R13-24 *G. beatrix* | MF038642 (COI); MF038541 (Cytb); MF038742 (12S) | X | X | - | Dzanga-Sangha, CAR | Hassanin et al. 2018 |
| *Glauconycteris beatrix* | EBD200205 *G. beatrix* (terra typica) | PZ458465 (COI); PV917313 (Cytb); PZ425908 (12S); PZ458438 (HDAC2); PZ458449 (STAT); PZ458473 (RIOK3); PZ458487 (COPS); PZ458505 (ACTP); PZ458521 (ZFYVE27_H1); PZ458522 (ZFYVE27_H2); PZ458531 (RAG2); PZ458550 (RODGI);  PZ468921 (ACOX2);  PZ468935 (ABHD11) | X | X | X | Mainland, Equatorial Guinea | This study, Torrent et al. 2025a |
| *Glauconycteris beatrix* | EBD220514 *G. beatrix* (terra typica) | PZ458466 (COI); PV917314 (Cytb); PZ425909 (12S); PZ458439 (HDAC2_H1); PZ458440 (HDAC2_H2); PZ458453 (STAT); PZ458474 (RIOK3); PZ458491 (COPS); PZ458509 (ACTP); PZ458523 (ZFYVE27_H1); PZ458524 (ZFYVE27_H2); PZ458532 (RAG2); PZ458554 (RODGI);  PZ468923 (ACOX2);  PZ468939 (ABHD11) | X | X | X | Mainland, Equatorial Guinea | This study, Torrent et al. 2025a |
| *Glauconycteris beatrix* | EBD230217 *G. beatrix* (terra typica) | PZ458452 (STAT); PZ458490 (COPS); PZ458508 (ACTP); PZ458553 (RODGI);  PZ468922 (ACOX2);  PZ468938 (ABHD11) | - |  | X | Mainland, Equatorial Guinea | This study |
| *Glauconycteris beatrix* | HNHM23262 *G. beatrix* | MF038641 (COI); MF038540 (Cytb); MF038741 (12S) | X | X | - | Nki NP, Cameroon | Hassanin et al. 2018 |
| *Glauconycteris beatrix* | 231125Gsp182 *G. beatrix* | PZ458470 (COI); PZ425899 (Cytb); PZ425913 (12S); PZ458442 (HDAC2); PZ458456 (STAT); PZ458477 (RIOK3); PZ458494 (COPS); PZ458512 (ACTP); PZ458527 (ZFYVE27); PZ458535 (RAG2); PZ458557 (RODGI);   PZ468925 (ACOX2) | X | X | X | Lobéké National Park, Cameroon | This study |
| *Glauconycteris humeralis* | MNHN2016-2802 *G. humeralis* | MF038597 (COI); MF038496 (Cytb); MF038697 (12S) | X | X | - | Sukisa, DRC | Hassanin et al. 2018 |
| *Glauconycteris lobeke* sp. nov. | R13-46 *G.* cf. *humeralis* | MF038598 (COI); MF038497 (Cytb); MF038698 (12S) | X | X | - | Dzanga-Sangha, CAR | Hassanin et al. 2018 |
| *Glauconycteris lobeke* sp. nov. | R13-98 *G.* cf. *humeralis* | MF038599 (COI); MF038498 (Cytb); MF038699 (12S) | X | X | - | Dzanga-Sangha, CAR | Hassanin et al. 2018 |
| *Glauconycteris lobeke* sp. nov. | 240522Gbe215 *G.* cf. *humeralis* | PZ458469 (COI); PZ425898 (Cytb); PZ425912 (12S); PZ458443 (HDAC2); PZ458457 (STAT); PZ458478 (RIOK3); PZ458495 (COPS); PZ458513 (ACTP); PZ458528 (ZFYVE27_H1); PZ458529 (ZFYVE27_H2); PZ458536 (RAG2); PZ458558 (RODGI);  PZ468920 (ACOX2);  PZ468942 (ABHD11) | X | X | X | Lobéké National Park, Cameroon | This study |
| *Glauconycteris lobeke* sp. nov. | 231119Gsp151 *G.* cf. *humeralis* | PZ458468 (COI); PZ425897 (Cytb); PZ425911 (12S); PZ458441 (HDAC2); PZ458455 (STAT); PZ458476 (RIOK3); PZ458493 (COPS); PZ458511 (ACTP); PZ458526 (ZFYVE27); PZ458534 (RAG2); PZ458556 (RODGI);  PZ468926 (ACOX2_H1);  PZ468927 (ACOX2_H2);  PZ468941 (ABHD11) | X | X | X | Lobéké National Park, Cameroon | This study |
| *Glauconycteris lobeke* sp. nov. | EBD190113Lo *G.* cf. *humeralis* | PZ458463 (COI); PV917324 (Cytb); PZ425906 (12S); PZ458436 (HDAC2); PZ458444 (STAT); PZ458471 (RIOK3); PZ458480 (COPS_H1); PZ458481 (COPS_H2); PZ458519 (ZFYVE27); PZ458544 (RODGI); PZ468918 (ACOX2); PZ468929 (ABHD_H1);  PZ468930 (ABHD_H2) | X | X | X | Mainland, Equatorial Guinea | This study, Torrent et al. 2025a |
| *Glauconycteris lobeke* sp. nov. | EBD190116 *G.* cf. *humeralis* | PZ458464(COI); PV917325 (Cytb); PZ425907 (12S); PZ458437 (HDAC2); PZ458472 (RIOK3); PZ458479 (COPS); PZ458520 (ZFYVE27); PZ458530 (RAG2); PZ458543 (RODGI);  PZ468919 (ACOX2);  PZ468928 (ABHD11) | X | X | X | Mainland, Equatorial Guinea | This study, Torrent et al. 2025a |
| *Glauconycteris baka* sp. nov. | 230718Gsp18 *G.* sp. nov. | PZ458467 (COI); PZ425896 (Cytb); PZ425910 (12S); PZ458454 (STAT); PZ458475 (RIOK3); PZ458492 (COPS); PZ458510 (ACTP); PZ458525 (ZFYVE27); PZ458533 (RAG2); PZ458555 (RODGI);  PZ468924 (ACOX2);  PZ468940 (ABHD11) | X | X | X | Lobéké National Park, Cameroon | This study |
| *Glauconycteris baka* sp. nov. | MNHN2016-2784 *G.* sp. nov. | MF038623 (COI); MF038522 (Cytb); MF038723 (12S) | X | X | - | Yatolema, DRC | Hassanin et al. 2018 |
| *Glauconycteris baka* sp. nov. | MNHN2016-2781 *G.* sp. nov. | MF038624 (COI); MF038523 (Cytb); MF038724 (12S) | X | X | - | Bongandjola, DRC | Hassanin et al. 2018 |
| *Glauconycteris baka* sp. nov. | MNHN2016-2787 *G.* sp. nov. | MF038630 (COI); MF038529 (Cytb); MF038730 (12S) | X | X | - | Yoko, DRC | Hassanin et al. 2018 |
| *Glauconycteris baka* sp. nov. | MNHN2016-2783 *G.* sp. nov. | MF038638 (COI); MF038537 (Cytb); MF038738 (12S) | X | X | - | Melume, DRC | Hassanin et al. 2018 |
| *Glauconycteris baka* sp. nov. | MNHN2016-2782 *G.* sp. nov. | MF038639 (COI); MF038538 (Cytb); MF038739 (12S) | X | X | - | Melume, DRC | Hassanin et al. 2018 |
| *Glauconycteris variegata* | EBD230213 *G. variegata* | PZ458451 (STAT); PZ458489 (COPS); PZ458507 (ACTP); PZ458552 (RODGI);  PZ468917 (ACOX2);  PZ468937 (ABHD11) | - | - | X | Mainland, Equatorial Guinea | This study |
| *Glauconycteris gleni* | 20242205222 *G. gleni* | PZ458539 (Cytb); PZ458498 (COPS_H1); PZ458499 (COPS_H2); PZ458516 (ACTP); PZ458561 (RODGI_H1); PZ458562 (RODGI_H2);  PZ468908 (ACOX2);  PZ468945 (ABHD11) | X | - | X | Lobéké National Park, Cameroon | This study |
