# Supplementary figures and images for: An integrated taxonomic and conservation assessment of Glauconycteris (Chiroptera, Vespertilionidae) in Cameroon, with the description of two new species from the Northwestern Congolian Lowland Forest
Source: Zookeys. 2026 Jun 15;1282:161–204. doi: 10.3897/zookeys.1282.183038 (PMC13288025; doi:10.3897/zookeys.1282.183038)

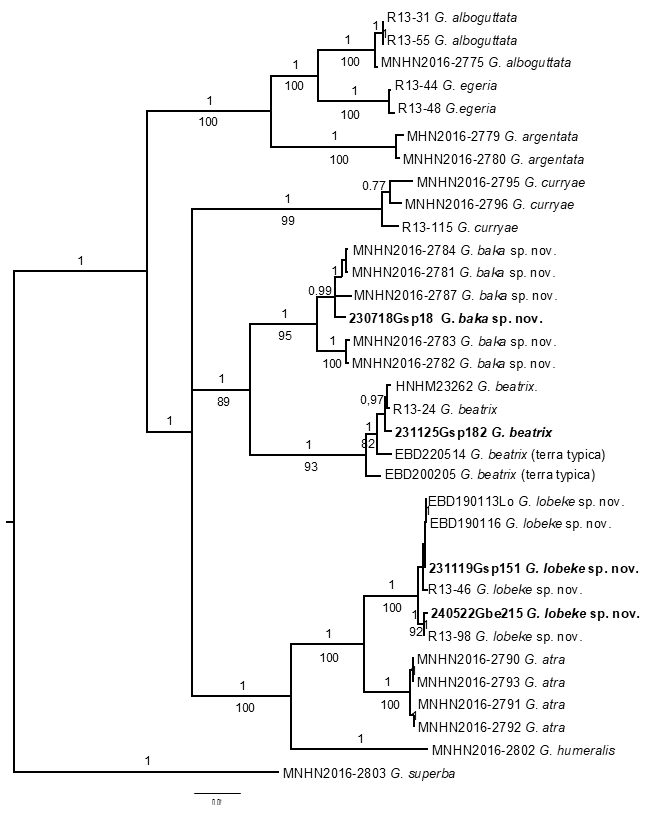

Supplement: Supplementary material 3 — Supplementary image 1 [file zookeys-1282-161_article-183038__-s003.png]

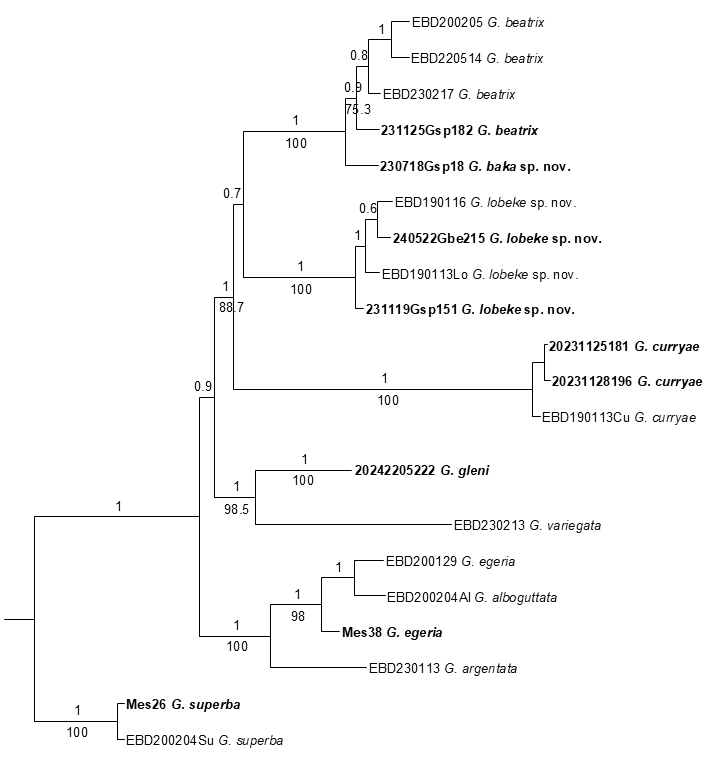

Supplement: Supplementary material 4 — Supplementary image 2 [file zookeys-1282-161_article-183038__-s004.png]
